# Angiotensin II prevents the metabolic but not the transdifferentiating effect of EGF on vascular smooth muscle cells from human female donors

**DOI:** 10.1016/j.isci.2026.115886

**Published:** 2026-06-02

**Authors:** Virginie Dubourg, Nasrin Akhtar, Michael Kopf, Amelie Spilker, Sigrid Mildenberger, Barbara Schreier, Gerald Schwerdt, Ronald Biemann, Ralf A. Benndorf, Michael Gekle

**Affiliations:** 1 Julius-Bernstein-Institute of Physiology, Martin Luther University Halle-Wittenberg, Halle (Saale), Germany; 2 Institute of Laboratory Medicine, Clinical Chemistry and Molecular Diagnostics, University of Leipzig, Leipzig, Germany; 3 Institute of Pharmacology and Toxicology, Ruhr University Bochum, Bochum, Germany

**Keywords:** Biological sciences

## Abstract

Vascular smooth muscle cells (VSMCs) are at the center of vascular diseases but studies on VSMCs from human females are rare and the medical need for female-specific vascular research is urgent. VSMCs are simultaneously subjected to paracrine and autocrine mediators, whose effects do not simply add up. For instance, a synergistic crosstalk occurs between the VSMC epidermal growth factor receptor (EGFR), a major transducer of autocrine signals (membrane-derived EGF), and the paracrine angiotensin II (AngII) type 1 receptor (AT1R). Here, we investigated the effects of EGF and AngII on primary human VSMCs from female donors. EGF initiates VSMC transdifferentiation toward a proliferative and inflammatory phenotype and affects glucose and lipid metabolisms. Co-exposure with AngII does not affect the EGF-induced transdifferentiation but counteracts the metabolic effects, independently of AT1R. Activation of the MAS1 receptor by angiotensin 1-7, an AngII-cleavage product, mimicked the AngII effects. Similar EGF-AngII interactions were not observed in male VSMCs.

## Highlights

• EGF induces VSMC transdifferentiation to a proliferative/inflammatory phenotype
• EGF affects VSMC glucose utilization and lipid accumulation
• Co-incubation with AngII influences EGF-induced metabolic changes only
• Such EGF-AngII interactions were observed in female but not in male VSMCs

## Graphical abstract

**Figure:**
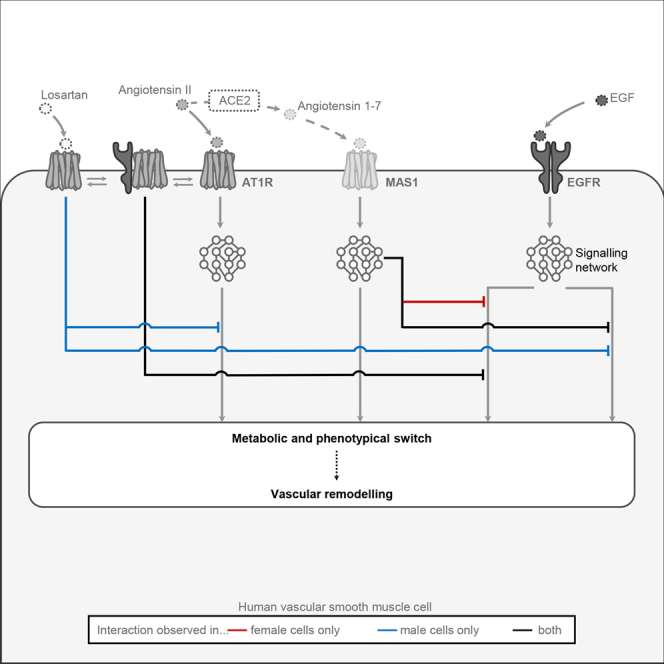

## Introduction

Vascular smooth muscle cells (VSMCs) are critical for the structure and function of blood vessels, particularly arteries. During vascular injury, VSMCs shift from a quiescent, contractile state to a more active, proliferative form, thereby contributing to vessel repair.^1^ However, it is crucial that VSMCs revert to their original, contractile state once the vessel homeostasis is restored. Indeed, a maintained de-differentiated state or a subsequent transition to an altered phenotype (e.g., foam-cell-, osteoblast-, or macrophage-like VSMCs) can result in VSMC-driven vascular diseases.^2^^,^^3^^,^^4^ This means that the dynamic nature of VSMCs not only shapes the physical characteristics of arteries but also influences the risk of developing associated disease.

Although it is established that women and men do not present the same cardiovascular risks and that some non-sex hormone-related differences between female and male VSMCs exist,^5^^,^^6^^,^^7^ in-depth study of human female VSMCs remain scarce. There is therefore an urgent need for vascular research focused on female vascular cells, in order to understand sex-specific aspects of the pathophysiology of vascular disease. This issue becomes even more pressing, when considering that common therapeutic drugs have sex-dependent efficacy or vascular side effects, as is the case for cancer therapies associated with cardiotoxicity.^8^ This also applies to inhibitors of the renin-angiotensin-aldosterone system (RAAS), which are commonly used as first-line treatment to overcome the hyperactivity of the RAAS in vascular disease and prevent the subsequent risks of, e.g., heart and kidney end-organ damages.^9^

Given this, we previously investigated the role of the epidermal growth factor (EGF) receptor (EGFR) in human primary female VSMCs. This receptor is best known as a driver of tumorigenesis and is the target of various anti-cancer therapies, which are partly associated with cardiotoxicity.^10^ But it also globally contributes to vascular physiology and pathophysiology, with reports showing increased EGFR expression in atherosclerotic plaques^11^ and patients with vascular calcification susceptibility,^12^ and an increased ligand availability (and therefore potentially enhanced EGFR activation) in patients with cardiovascular conditions.^13^^,^^14^^,^^15^ Our previous results showed that EGFR activation altered the human primary female VSMC transcriptome, putatively resulting in a transdifferentiation toward a proliferative and inflammatory phenotype.^16^ They were thus consistent with the idea that EGFR is critical in cardiovascular pathology development, including in women.

However, *in vivo* cells are simultaneously subjected to different mediators, whose combined effect is not equal to the simple addition of the individual effects. For instance, besides the activation of the EGFR by autocrine signals (membrane-derived EGF), other receptors such as the angiotensin II (AngII) type 1 receptor (AT1R) can integrate paracrine signals. But cross-activation between these two processes also occur, since EGFR is a signaling hub that get indirectly activated (or “transactivated”) by receptors for vasoactive substances like AT1R.^17^ EGFR thereby also contributes to the impact of AngII-associated signaling pathways (and therefore of RAAS) on vasculature, as shown in mouse models where EGFR knockout prevents AngII-induced hypertension, arterial wall stiffening, and media thickening.^18^^,^^19^^,^^20^^,^^21^ Using stably AT1R-transfected HEK293 cells to characterize further underlying mechanisms, we showed that an AT1R-EGFR crosstalk leads to a synergistic time-dependent regulation of gene expression,^22^ which requires the physical interaction of both receptors.^23^ Moving from this experimental model to primary cells, we confirmed a temporal synergy of EGF and AngII in murine VSMCs.^24^

Here, to translate these results to human pathophysiology, we investigated how EGF and AngII influence human primary VSMCs, using primarily cells from female donors. In addition to the need of sex-differentiating research mentioned hereinabove, considering both sex groups separately for these research questions was a necessity, given that EGFR and AT1R expression and activity levels vary between males and females^25^^,^^26^^,^^27^ and that therapies targeting them have different efficacy in both sex groups.^28^^,^^29^ We thus (1) tested phenotypical traits on which we assumed EGF had an effect based on prior knowledge (e.g., inflammation and proliferation) and (2) applied an untargeted transcriptomic approach to identify other cellular functions whose regulation by EGF can be modulated by AngII. Selected experiments were repeated in male cells to define if the observed effects were sex specific or not. This strategy revealed new aspects of the crosstalk between these two vasoactive substances that may play a critical role in female blood vessels.

## Results

### *Ex vivo* human female VSMCs, an appropriate model to investigate EGF and AngII functional interaction

We assessed the expression levels of EGF and AngII receptors and their associates using RNA-sequencing data (Figure S1A). Among ERBB family members, EGFR (ERBB1) and ERBB2 were predominant. Concerning AngII receptors, AT1R was lowly expressed, and its counterpart AT2R was not detectable, like many G-protein-coupled receptors associated with cardiovascular functions^30^ (Figure S2). MAS1, the receptor for natural AngII cleavage product, angiotensin 1-7 (Ang1-7), was detected. In the absence of specific antibodies, their expression levels were confirmed at mRNA level (Figures S1B–S1D).

Because AngII receptors were lowly expressed, the responsiveness of VSMCs to AngII was tested by measuring the phosphorylation level of ERK1/2, a major player in AngII-induced pathways, at different AngII concentrations. AngII induced an increase in ERK1/2 phosphorylation from 1 nM (Figure S1E), despite the high basal ERK1/2 phosphorylation level of VSMCs (Figures S1F and S1G). Losartan, an AT1R-blocker, prevented this effect (Figure S1H). This means that VSMCs respond to the physiopathological relevant AngII concentration (10 nM)^22^ used here, including via the AT1R axis. *Ex vivo* primary human VSMCs are thus an appropriate study model for the study of EGF and AngII functional interaction.

### Cell proliferation is affected by EGF but without additional effect of AngII

We previously hypothesized that EGF induces VSMC transdifferentiation, toward a less contractile but more proliferative and inflammatory phenotype.^16^ We therefore began by testing whether EGF affected these aspects. We also tested the effect of AngII and whether it could modulate that of EGF when combined. Both did not cause deleterious effects (Figures S3A and S3B), meaning that further effects are unlikely to result from non-specific cell death.

**Figure 1:**
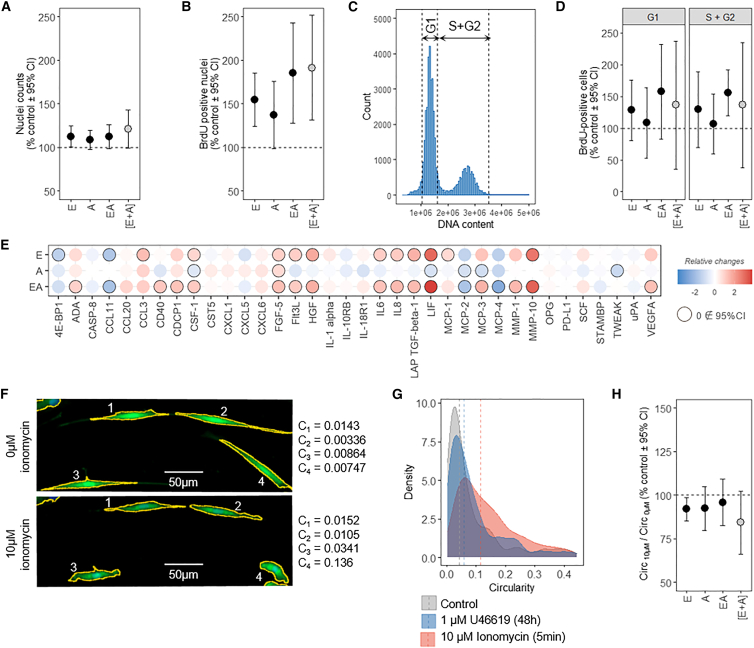
EGF initiates a transdifferentiation of female human VSMCs toward a more proliferative and inflammatory phenotype (A) Cell proliferation was estimated by counting the number of stained nuclei after 48-h incubation with EGF and/or AngII (same initial number of cells for each condition) (*N* = 14, 3 donors). (B) DNA synthesis was measured by BrdU incorporation, and BrdU-positive cells (nuclei whose red fluorescence was above the background) were counted (*N* = 9, 3 donors). (C) The distribution of DNA content of the individual nuclei allowed the distinction of those in the G1 phase of the cell cycle or in the S and G2 phases (combined, noted S + G2). (D) The number of BrdU-positive cells per cell-cycle phases was counted. (E) Secreted inflammation markers and their relative changes compared to control conditions (*N* = 7, 2 donors). (F) Cell circularity was used as a proxy for cell shape measurement. Here are shown some examples of VSMCs before (upper picture) and after (lower picture) incubation with ionomycin, an ionophore that induces an increase in intracellular calcium concentration and therefore a contraction. The yellow lines were drawn by the Gen5 software and show how the cells were recognized. The circularity C of the different objects is indicated. The scale bars correspond to 50 μm. (G) Distribution of the circularity for cells kept under different conditions. The dotted lines correspond to the respective medians. (H) The amplitude of the Ca^2+^-dependent contraction is returned by the ratio of the cell circularity with ionomycin and the initial one (*N* = 14, 3 donors). E, EGF; A, AngII; EA, EGF and AngII; [E + A], calculated expected additive effect for EGF and AngII. The dotted lines in the figures with mean ± CI correspond to the reference level (control values set at 100% for each independent biological replicate). Effects were considered statistically significant when the CI did not include the reference value. See also Figure S3.

Cell proliferation was measured by counting the number of cells/nuclei after 48 h (Figure 1A). EGF resulted in a seemingly modest increase in nuclei counts (113% ± 12%). However, the increase caused by fetal calf serum (FCS) and growth factors (positive control) was only of 125% (±14%) (Figure S3C), underscoring the relevance of small EGF-induced changes. We also quantified the amount of synthesized DNA by measuring BrdU incorporation, a more sensitive approach as evidenced by the clear FCS-induced signal increase (831% ± 308, Figure S3D). EGF induced an increase in BrdU-positive nuclei (Figure 1B). Although statistical significance was not reached, AngII also tended to increase the number of nuclei and BrdU-positive nuclei (Figures 1A and 1B). However, the effect of the combined stimuli was indistinguishable from the calculated expected additive effect (denoted [E + A]), suggesting an absence or weak functional interaction of the two substances regarding proliferation. A more detailed approach reporting the number of cells and BrdU-positive cells in the different phases of the cell cycle led to the same conclusion (Figures 1C and 1D; Figure S3E).

### EGF and AngII influence the secretion of inflammatory markers

VSMC inflammation profiles were investigated by measuring secreted markers. Out of 92 measured markers, 36 were detectable in at least one condition. Thirteen and six markers appeared regulated by EGF or AngII, respectively (Figure 1E; Table S1). Each substance can therefore modulate inflammation. EGF regulated typical pro-inflammatory markers such as interleukin (IL)-6, IL-8, and transforming growth factor β1 (TGF-β1) precursor. The co-stimulation had a similar effect to that of EGF alone, except for a few markers like ADA (adenosine deaminase), CD40, or VEGFA (vascular endothelial growth factor A).

### EGF impairs VSMC Ca^2+^-dependent contractility potential

Based on these indications of an EGF-induced phenotypical switch, we investigated whether VSMC contractility potential was altered. To do so, we measured cell circularity (cell shape indicator) and assessed how contraction-inducing substances changed it. This approach was first validated with VSMCs grown under control conditions. Cell contraction (measured here by an increase in circularity) was visible following acute incubation with the Ca^2+^-ionophore ionomycin (Figures 1F and 1G). A 48-h incubation with U46619 (thromboxane A2 analogue) also showed that VSMCs can maintain a certain contracted state (Figure 1G; Figure S3G). The same procedure was repeated on cells pre-incubated with EGF or/and AngII. They had a similar initial cell shape, and all contracted to some degree (Figure S3G and S3H). But EGF-incubated cells showed a slightly reduced responsiveness to ionomycin (Figure 1H). The variability and low amplitude of the effect preclude any conclusion as to whether AngII influences this effect, although AngII did not affect known EGF-regulated differentiation markers^16^ (Figures S3I–S3K). Overall, these results indicate that EGF initiates a phenotypical switch in female VSMCs, probably independently of AngII.

### AngII modulates EGF-induced transcriptomic changes

**Figure 2:**
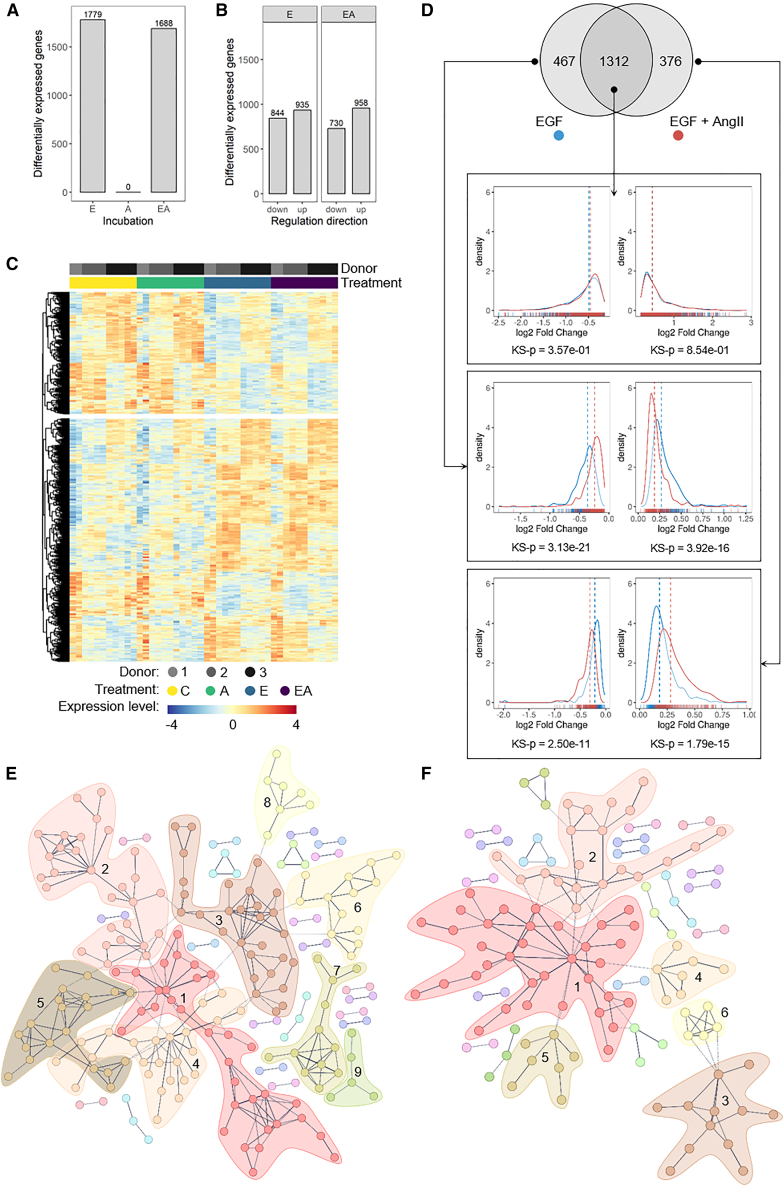
AngII affects the EGF-induced gene expression regulation in VSMCs (A) Number of differentially expressed genes identified for each of the condition (defined as gene with an false discovery rate (FDR) below 0.05 in both DESeq2 and edgeR outputs and with at least five FPM on average in one of the sample groups). (B) Number of up- and down-regulated genes for each of the conditions that led to any gene expression regulation in the first place. (C) Heatmap showing the normalized expression (log scale) of the 2,155 genes identified as significantly regulated for at least one comparison (EGF alone or with AngII vs. control group). Each row corresponds to a gene and each column to a sample. Expression levels were additionally row-wise centered (subtraction of the mean from each value) and scaled (division by the standard deviation). Rows were clustered based on Euclidean distance (complete method). (D) Overlap of the lists of genes regulated by EGF or by EGF combined with AngII. Lower panels display the distribution of the log2 fold changes of the genes comprised in the different parts of the Venn diagram. Kolmogrov-Smirnov testing was used to evaluate the relevance of the observed shifts (*p* values are indicated on the figure). Clustering of the protein-coding genes regulated exclusively (E) by EGF alone or (F) by the combination of EGF and AngII (correspond to the outer parts of the Venn Diagram in D). For each, clusters with more than three nodes were highlighted, and the corresponding information have been listed in Table 1 (complete data in Table S2). C, control; E, EGF; A, AngII; EA, EGF + AngII.

An RNA-sequencing-based non-targeted approach was used to identify additional aspects potentially impacted by the crosstalk between EGF- and AngII-associated pathways. Differential expression analysis showed that AngII alone did not lead to any measurable changes in gene expression (Figure 2A), although VSMCs responded to this substance (Figure S1E; Figure 1). Conversely, EGF itself or in combination with AngII led to a substantial number of regulated genes (Figure 2A; Table S2). The addition of AngII did not alter the absolute number of regulated genes compared with EGF alone (Pearson’s chi-squared test *p* value = 0.11) but shifted the regulation direction (more down- than up-regulated genes; *p* = 1.44 × 10^−8^) (Figure 2B).

Remarkably, although the numbers of regulated genes were similar when comparing the effect of EGF alone or in combination with AngII, the portfolios of regulated genes partly changed (Figures 2C and 2D). One thousand three hundred twelve genes were regulated in both cases. Their regulation amplitudes suggest that EGF alone already led to the maximal response and that AngII supplementation did not have any additional effect (Figure 2D). Meanwhile, over 20% of the genes regulated by one incubation type were specific to it (outer parts of the Venn diagram). The dependency of the regulation amplitude of these genes on the type of incubation confirms that both types of incubation partially drive differential transcriptomic responses (Figure 2D).

### The qualitative synergism of EGF and AngII results in a functional shift

Clustering of the incubation-type-specific regulated protein-coding genes further support this hypothesis as different functionally coherent clusters were identified (Figures 2E and 2F; Table 1; Table S2), including clusters associated with glucose and cholesterol metabolisms.

**Table 1:** Regulated protein-coding genes cluster in functional groups

Treatment	Cluster	Genes	Description	
EGF	1	33	assembly of collagen fibrils and other multimeric structures, protein digestion and absorption, extracellular matrix structural constituent	
	2	29	–	
	3	29	preribosome and ribosome biogenesis	
	4	24	RAC3 GTPase cycle	
	5	20	platelet activation, opioid signaling	
	6	13	autophagosome assembly, amino acids regulate mTORC1, and TORC2 complex	
	7	12	cholesterol biosynthesis	(L)
	8	7	glucose 6-phosphate metabolism, pentose phosphate pathway	(G)
	9	5	NLS-bearing protein import into nucleus, Ran-binding domain, nucleocytoplasmic carrier activity, and Importin-beta, N-terminal domain	
EGF + AngII	1	35	–	
	2	20	positive regulation of endothelial cell proliferation, cell junction organization	
	3	12	–	
	4	7	PRC2 methylates histones and DNA, replication fork	
	5	7	dicarboxylic acid metabolism, purine biosynthesis, citrate cycle (TCA cycle), and lactate dehydrogenase activity	(L)/(G)
	6	5	CENP-A containing nucleosome, structural constituent of chromatin, histone H4	

Functions were associated with the different clusters identified by STRING (Figures 2E and 2F), whose listed descriptions (from primary to tertiary descriptions if available) correspond to enriched Gene Ontology terms or pathways. Clusters associated with lipid- or glucose-related metabolism are marked with (L) and (G), respectively. The complete lists of cluster (including those with fewer nodes) are included in Table S2.

**Figure 3:**
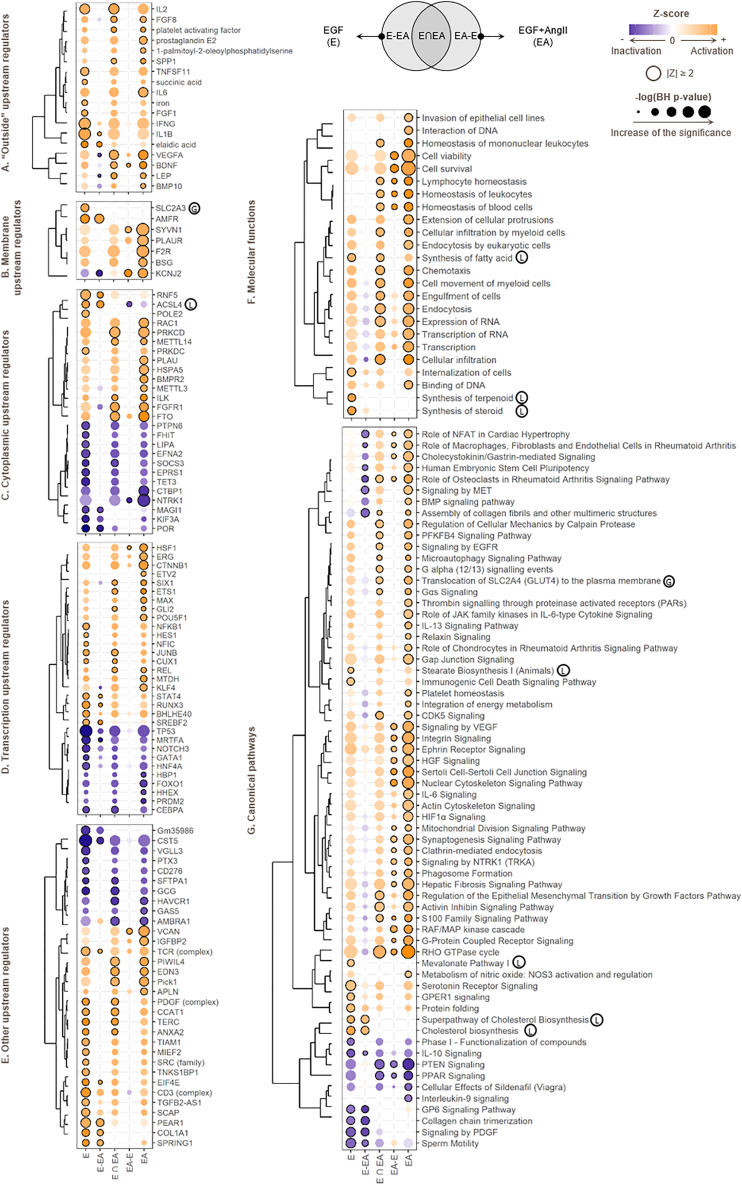
The combination of EGF with AngII induces a functional shift compared to its single effect In order to identify cellular mechanisms and cellular outputs possibly triggered in an incubation-type-specific manner, the predicted upstream regulators of gene expression regulation and affected downstream cellular functions or pathways were filtered and divided into five layers (see Methods). For each of the five upstream- (A–E) and two downstream sections (F and G), different conditions are compared. E and EA correspond to all genes regulated by EGF alone or combined with AngII, respectively. E-EA, E∩EA, and EA-E correspond to the genes comprised in the different groups identified in the Venn diagram of Figure 2D. Terms directly related to lipid or glucose metabolisms are marked with “L” and “G”, respectively. The complete data are included in Table S2.

Differential functional enrichment analysis with ingenuity pathway analysis (IPA) also predicted such outcomes (Figure 3; Table S2). Pathways and molecular functions showed incubation-type-specific regulation patterns (Figures 3F and 3G). Those comprised terms associated with, e.g., inflammation, extracellular matrix, or glucose and lipid metabolism. Similarly, upstream regulators predicted in both scenarios differed (Figures 3A–3E). For example, we can pin-point ACSL4 (acyl-CoA synthetase long chain family member 4), a key player in lipid metabolism, or SLC2A3 (glucose transporter type 3), which were predicted to be more active in presence of EGF alone only.

**Figure 4:**
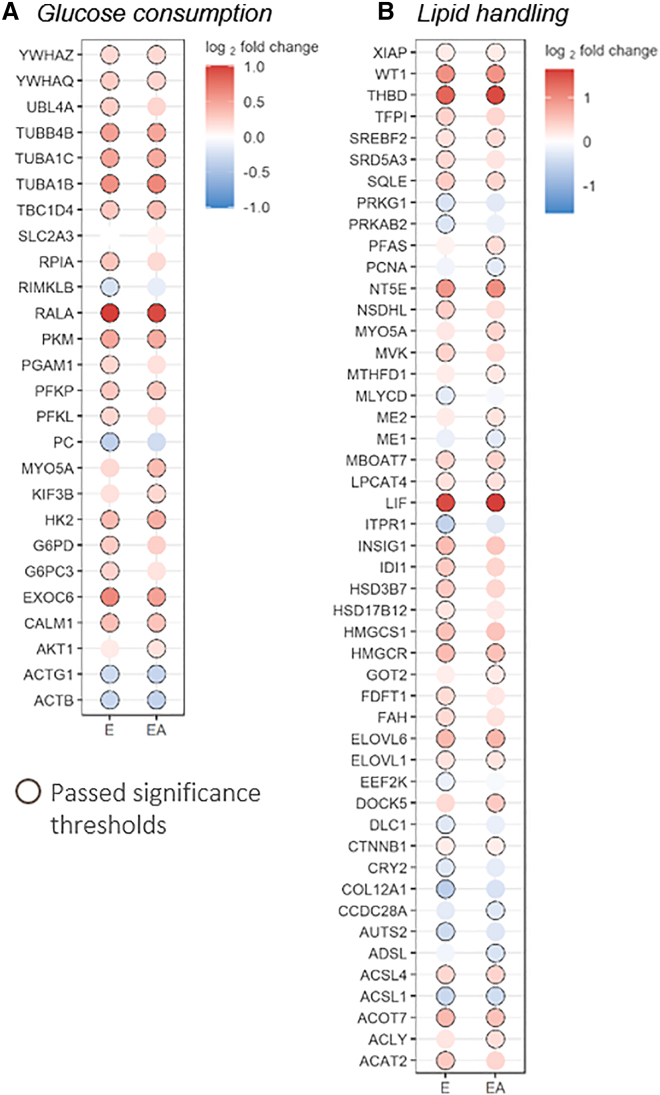
EGF and EGF + AngII differentially regulate genes associated with lipid and glucose metabolism The expression patterns of the genes included in the (A) glucose and (B) lipid-related clusters or functions (Table S2; Figure 3G) are displayed here. Log_2_ fold changes calculated by DESeq2 were used for visualization. The significance thresholds are defined in the method section.

Therefore, co-stimulation with EGF and AngII not only leads to a qualitative synergy by inducing a partial change in the regulated gene portfolios, but may also change the regulated pathways and cellular responses of VSMCs. More specifically, we further hypothesized that the effect of the combination of EGF and AngII on VSMC metabolism differ from the one caused by EGF itself based on different enrichment for related pathways and on the differential regulation patterns of the included genes (Figure 4).

### AngII prevents the EGF-induced effect on VSMC glucose and lipid handling

**Figure 5:**
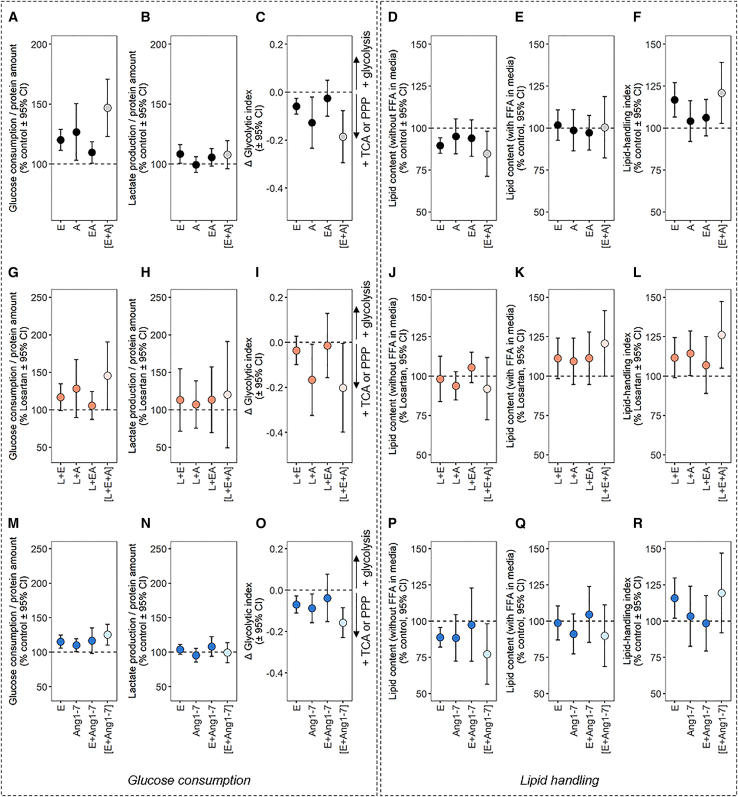
AngII prevents the effect of EGF on glucose and lipid handling in female VSMCs (A) Glucose consumption and (B) lactate production by VSMCs were measured. (*N* = 12, 3 donors). (C) Changes concerning the glycolytic state of the cells (represented here by ΔGlycolytic index) were defined as the difference between the glycolytic indexes of the cells with a given treatment and under control conditions (Figure S4A, Methods). The lipid content of VSMCs was measured for each treatment condition, (D) without or (E) with free fatty acids (FFAs) in the cell culture media. (*N* = 19, 3 donors). (F) The overall ability of the cells to use FFA was calculated as the ratio of the amount of intracellular lipids with and without fatty acids in the media. Using the same approach, the influence of losartan, an AT1R inhibitor, on EGF and AngII effects was investigated regarding (G–I) glucose metabolism (*N* = 7, 2 donors) and (J–L) lipid content (*N* = 11, 2 donors). The same experiments were repeated to investigate the interaction of EGF with Ang1-7 on (M–O) glucose metabolism (*N* = 9, 2 donors) and (P–R) lipid metabolism (*N* = 11, 2 donors). C, control; E, EGF; A, AngII; EA, EGF and AngII; [E + A], calculated expected additive effect for EGF and AngII; L, losartan; [L + E + A], calculated expected additive effect for combined losartan, EGF, and AngII; [E + Ang1-7], calculated expected additive effect for EGF and Ang1-7. The dotted lines in the figures displaying means ± 95% confidence interval (CI) correspond to the reference level (values corresponding to control or losartan-alone conditions were set at 100% for each independent biological replicate). See also Figure S4.

We first measured glucose consumption and lactate production and calculated a glycolytic index (produced lactate/2 × consumed glucose) to estimate how much glucose was consumed though glycolysis. Under control conditions, cells had a glycolytic index below one, meaning that they did not convert all glucose to lactate (Figure S4A). EGF led to an increase in glucose consumption and lactate production, but not in a proportional manner since the glycolytic index decreased further, suggesting it promoted the tricarboxylic acid (TCA) cycle or pentose phosphate pathway (PPP) over glycolysis-related lactate production (Figures 5A–5C; Figure S4A). AngII led to an increase in glucose consumption without changing the lactate production, also inducing a metabolic shift. However, these effects antagonized each other to some extent regarding glucose consumption and completely regarding the glycolytic index, as seen when comparing the measured and expected additive values of the co-incubation (EA vs. [E + A]; Figures 5A–5C; Figure S4A).

Additionally, the neutral lipid content of the cells was measured under two conditions. First, it was assessed after incubation with EGF and/or AngII, without adding free fatty acid (FFA) to the media (Figure 5D). This allowed observing the effect of the hormones on basal neutral lipid homeostasis. EGF incubation reduced it. Second, the experiments were also performed with the addition of FFA to the media (Figure 5E), thereby checking if EGF and/or AngII modulate lipid accumulation under conditions of high FFA abundance. This was done to unveil a possible effect of the substances when cells are forced to accumulate lipids. In that case, EGF did not change the lipid content of cells. The lipid-handling index (defined as the amount of neutral lipids in cells supplemented with FFA divided by the amount of neutral lipids in cells without FFA-supplementation) was thus increased by EGF (Figure 5F), suggesting that EGF-treated cells have overall an enhanced ability to use lipids. AngII did not affect the lipid-related parameters but still prevented that of EGF when both were combined (Figures 5D–5F; Figure S4D).

### AngII does not modulate the effect of EGF on metabolism via its canonical AT1R pathway

We investigated whether the interaction between EGF and AngII depended on the canonical AT1R signaling pathway or an alternative pathway involving AT2R or MAS1. AT2R was not detectable but MAS1 was (Figure S1C), as well as the enzymes ACE and ACE2 that are required to cleave AngII and generate MAS1-ligand (Figure S5A). The measurements regarding glucose and lipid metabolisms were repeated in the presence of (1) losartan to see if we could prevent the interaction of EGF and AngII and (2) Ang1-7 to test if the specific activation of the MAS1 pathway had similar outcomes as AngII incubation.

Losartan did not prevent the impact of AngII on glucose handling but prevented that of EGF (Figures 5G–5I; Figure S4B). Concerning lipid-related parameters, losartan had an effect on its own, both without and with FFA in the media (Figure S4E), thereby leading to a dampening of the substance-induced effects compared to the initial experiments. This ultimately prevented the EGF-induced effect on basal lipid homeostasis (Figures 5D–5J). To assess if AT1R blockade by losartan could prevent other AngII effects and metabolism-unrelated EGF effects, cell proliferation was measured. Losartan also did not prevent the AngII-induced increase in nuclei count, and the nuclei count increased in the presence of EGF and losartan, to a similar extent to when EGF was alone (Figure S5B; Figure 1A). These results suggest that AngII acts on female VSMCs in an AT1R-independent manner and that losartan only partly affects EGF downstream effects.

Like AngII, Ang1-7 led to an increased glucose consumption but did not influence lactate production, leading to a reduced glycolytic state (Figures 5M–5O; Figure S4C). The combination of EGF and Ang1-7 prevented the EGF-induced glycolytic shift (Figure 5O; Figure S4C). Moreover, Ang1-7 had little effect on lipid handling but its addition counteracted that of EGF (Figures 5P–5R; Figure S4F). Therefore, the results show a negative interaction between Ang1-7 and EGF regarding metabolic traits, similar to the one observed between AngII and EGF. An inhibitory effect of Ang1-7 over EGF was also observed regarding cell proliferation but not cell contractility (Figures S5C–S5E).

### AngII and EGF functional interaction does not impact male VSMC metabolism

In order to assess if the functional interaction of AngII and EGF regarding metabolic traits was a sex-specific feature, glucose consumption, lactate production, and neutral lipid content were also measured in male VSMCs. Cell proliferation and contractility were measured for these cells as well.

Male VSMCs also express EGFR and AT1R, albeit at low level compared to female VSMCs (in average 2- and 5-fold less, respectively) (Figures S1 and S6). EGF and AngII led to an increase in cell proliferation of male VSMCs as in female ones, but these effects were prevented by losartan in male cells (Figures S7A and S7B). This increased proliferation was associated with a decrease in ACTA2 expression (contractility marker) and an increase in EGR1 expression (proliferative marker) (Figures S7C and S7D), without nonetheless leading to a measurable change in Ca^2+^-dependent contractility potential (Figure S7E).

**Figure 6:**
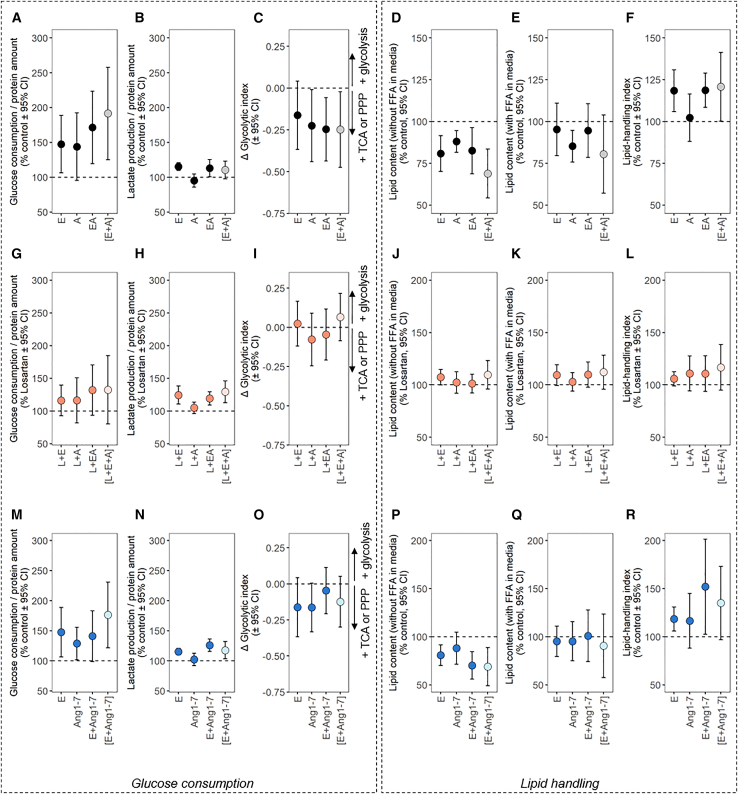
AngII does not modulate the effect of EGF on male VSMC metabolic traits Glucose consumption (A) and lactate production (B) by male VSMCs were measured (*N* = 10, 2 donors). (C) Changes concerning the glycolytic state of the cells was defined as the difference between the glycolytic index (Figure S4G) of the cells with a given treatment and under control conditions. The lipid content of VSMCs was measured for each treatment condition, (D) in the presence or (E) not of FFA in the cell culture media. (*N* = 13, 2 donors). (F) The overall ability of the cells to use FFA was calculated as the ratio of the amount of lipids with and without fatty acids in the media. Using the same approach, the influence of losartan, an AT1R inhibitor, on EGF and AngII effects was investigated regarding (G–I) glucose metabolism (*N* = 10, 2 donors) and (J–L) lipid content (*N* = 12–13, 2 donors). The same experiments were repeated to investigate the interaction of EGF with Ang1-7 on (M–O) glucose metabolism (*N* = 10, 2 donors) and (P–R) lipid metabolism (*N* = 13, 2 donors). C, control; E, EGF; A, AngII; EA, EGF and AngII; [E + A], calculated expected additive effect for EGF and AngII; L, losartan; [L + E + A], calculated expected additive effect for combined losartan, EGF and AngII, [E + Ang1-7] = calculated expected additive effect for EGF and Ang1-7. The dotted lines in the figures displaying means ± 95% CI correspond to the reference level (values corresponding to control or losartan-alone conditions were set at 100% for each independent biological replicate). See also Figure S4.

In male VSMCs, EGF or AngII led also to an increase in glucose consumption and to a switch in favored metabolic pathways (Figures 6A–6C; Figure S4G). Regarding parameters associated with lipid handling, EGF had the same effect as in female cells, while AngII reduced the neutral lipid content of male VSMCs only, independently of the FFA presence in the media (Figures 6D–6F). As observed in female cells, losartan prevented the effect of EGF on these parameters. But unlike in female cells, the metabolism-related AngII effects were prevented by AT1R-blockade (Figures 6G–6L), suggesting that AngII acts via AT1R in male cells. Finally, the combination of EGF and AngII did not prevent their individual effects on these metabolic traits, and the measured additive effects did not differ from the expected calculated ones (EA vs. [E + A]; Figures 6A–6F). The combination of EGF and Ang1-7 also did not prevent the effects of EGF on metabolic traits, although it did prevent the EGF-induced increased cell proliferation (Figures 6M–6R; Figure S7F). In essence, no functional interaction between EGF and AngII took place in male VSMCs regarding glucose and lipid handling.

## Discussion

The present study was organized around two major axes. First, we aimed to test our previously formulated hypothesis that EGF induces a phenotypical switch in female human VSMCs, and additionally we tested if AngII could influence this outcome. The second part was based on transcriptome data and bioinformatics analyses and consisted in identifying additional functional aspects in which EGF and AngII pathways interacted. These two different aspects will be discussed successively.

### EGF initiates a *trans*-differentiation in female human VSMCs

Our results show that EGF induces proliferation, changes in inflammation profiles, and a decrease in Ca^2+^-dependent contractility, validating our previous hypothesis that EGF induces a phenotypical switch in female human VSMCs.^16^ This phenotypical switch observed here thus consists of a transition from a contractile and quiescent phenotype to a less contractile but more proliferative and inflammatory phenotype. The effect of EGF on cell proliferation is well established. Conversely, there is still little evidence of its involvement in the inflammatory response of vascular cells.^31^^,^^32^ The screening approach used here revealed that EGF induces the regulation of pro-inflammatory markers such as interleukin (IL)-6 and IL-8, whose elevated serum levels have been associated with the development or poor outcome of cardiovascular disease.^33^^,^^34^ Additionally, the two markers showing the strongest regulation (at least 3-fold increase) were the IL-6 class cytokine LIF (leukemia inhibitory factor) and the matrix metalloproteinase MMP-10, which have both been associated with vascular calcification.^35^^,^^36^ Thus, we report here further evidence that EGF regulates the secretion of inflammation mediators by vascular cells that may contribute to vascular diseases. In addition, the down-regulation of contractile phenotype markers and a reduced Ca^2+^-dependent contractility suggest that EGF affects VSMC contractile machinery. Overall, EGF triggers a critical transdifferentiation of female VSMCs toward a more proliferative and inflammatory phenotype, what can ultimately result in vascular dysfunction.

On the other hand, our results show that the addition of AngII does not affect this transdifferentiation effect induced by EGF. However, we must qualify this conclusion. Indeed, all experiments were performed after 48 h of stimulation, and we cannot currently rule out the possibility that an interaction between EGF and AngII regarding proliferation and inflammation occurs at other time points. This question will be addressed later in order to determine whether there is in fact a temporal pattern. Nevertheless, the 48-h time point was chosen because we wanted to identify early changes in cell phenotype induced by sustained increases in EGF and AngII concentrations, as seen in cardiovascular pathologies. We note that at this stage, the determining factor in pro-proliferative and pro-inflammatory changes in VSMC phenotype is EGF.

### Qualitative interaction of EGF and AngII regarding gene expression regulation in female human VSMCs

To identify functional synergisms of EGF and AngII, we generated a new RNA sequencing dataset, containing over 10 independent replicates, with cells from different donors harvested over several passages. Despite its robustness, this dataset did not permit to identify AngII-induced transcriptome changes, although the cells were AngII-responsive (regarding proliferation, inflammation, or ERK1/2-phosphorylation). While surprising, it confirms our previous observations showing a transitory effect of AngII on gene expression, with a peak occurring in earlier time points than the 48-h snapshot used here.^22^^,^^24^

Unlike in AT1R-transfected HEK293 cells and murine VSMCs,^22^^,^^24^ the combination of EGF and AngII did not result in a striking quantitative over-additive gene expression regulation. Different aspects explain it. First, while they constitute a useful tool to study signaling networks, AT1R-transfected HEK293 cells are an experimental overexpressing model that are not adequate to predict relevant phenotypical outcomes of primary, differentiated cells. Second, human and mice have different AngII-receptor panels, with mice having an additional AT1R isoform (AT1R_A_ and AT1R_B_), which may partly lead to different outcomes.^37^ EGF and AngII regulated genes related to proliferation and differentiation in both organisms, demonstrating the validity of transposing certain effects from mice to humans, but subtler effects concerning functions important to human health may have been overlooked in murine cell experiments. Thus, our data emphasize again the necessity of studies with primary human cells to identify effects and mechanisms that cannot be translated from other model systems.

Although AngII did not influence the amplitude of the transcriptome response to EGF in female human VSMCs, it did affect the identity of the genes regulated. Following different steps of functional analysis, we hypothesized that AngII modulates the effect of EGF on glucose and lipid handling in these cells.

### The effect of EGF on glucose utilization by female human VSMCs is prevented by AngII

VSMCs showed a high glycolytic rate under control conditions (at near physiological pO_2_), indicating that most of the consumed glucose was converted into lactate (even if we cannot exclude here that the lactate partly originates from glutamine). This high aerobic glycolysis rate is a known feature for VSMCs and may be necessary for specific cellular processes or to constitute a mitochondrial reserve capacity.^38^ EGF or AngII alone disrupted this balance. In both cases, less glucose was converted into lactate and more was fed into other pathways, like the TCA cycle, the PPP, or the serine and hexosamine biosynthetic pathways. This shift appeared particularly relevant since it was suggested previously that the phenotypical switch of VSMCs is driven by a metabolic switch.^38^ This effect was nonetheless prevented by the combination of EGF and AngII.

The control of cancer cell metabolism by EGFR has been reviewed but its role in vascular cell metabolism remains vague.^39^ Results obtained with the A7r5 cell line, a rat smooth muscle model, already suggested that EGF modulates the pathways by which cells utilize glucose: EGF induced a down-regulation of their glycolytic index and increased mitochondrial respiration.^21^ Our results are consistent with the above and provide initial evidence of an EGF effect on glycolytic processes in human VSMCs.

In our RNA sequencing dataset, EGF alone or with AngII leads to upregulation of elements crucial for glycolysis (e.g., hexokinase 2 [HK2], pyruvate kinase M1/2 [PKM], and phosphofructokinase platelet [PFKP]). But only EGF alone up-regulates PFKL (phosphofructokinase liver), another isoform of PFK1 (phosphofructokinase, converts fructose-6-phosphate to fructose-1,6-biphosphate). PFK1 isoforms have different substrate affinity and enzyme activity, resulting in different glucose consumption efficiencies.^40^ A change in their distributions could partly explain the higher glucose consumption with EGF compared with EGF + AngII. Furthermore, EGF alone also up-regulated genes encoding PPP key enzymes, like G6PD (glucose-6-phosphate dehydrogenase) and RPIA (ribose-5-phosphate isomerase A). This suggests that EGF also promotes glycolysis-branched pathways such as PPP. The latter contributes to nucleotide and therefore DNA synthesis. But the promotion of PPP has also been associated with VSMC phenotypical switch and cardiovascular pathological situations.^41^^,^^42^ Consequently, the prevention of altered glucose metabolism by the combination of EGF and AngII could be beneficial overall.

### AngII also prevents the effect of EGF on lipid handling by female human VSMCs

We measured VSMC neutral lipid content, with or without FFA in medium. Despite the complexity of lipid metabolism, and although more refined methods are required to fully understand the extent of the changes observed, this approach gave us a first rough estimate of the VSMC lipid handling capabilities. EGF leads to a decrease of the lipid content when cells are kept under FFA-free conditions (but not in the presence of a virtually unlimited source of FFA). It has been documented that synthetic VSMCs, a condition close to that caused by EGF, exhibit increased fatty acid oxidation, which is the process by which neutral lipids are consumed.^43^ EGF-induced transdifferentiation could therefore associate with increased lipid consumption. Since the pathway analysis also predicted an EGF-induced increase in cholesterol and other lipid synthesis, EGF may increase lipid consumption while inducing a compensatory increase in their synthesis to ensure increased lipid turnover. However, EGFR activation was also associated in intestinal colorectal adenocarcinoma cells with a change in fatty acid metabolism, incorporating them into polar rather than neutral lipids.^44^ If a similar process occurred in VSMCs, the decrease in neutral lipid content observed here could be due to a resetting of the lipid utilization pathways. The distinction between EGF-induced enhanced lipid consumption and changes in lipid-related metabolic pathways will require further investigation.

AngII did not affect the lipid content of the cells but still prevented the effect of EGF. This may be explained by the differential regulation of key players in lipid metabolism: EGF alone up-regulated, e.g., ACAT2 (cytosolic acetoacetyl-CoA thiolase enzyme), MVK (mevalonate kinase), INSIG1 (insulin-induced gene 1), and IDI1 (isopentenyl-diphosphate delta isomerase 1), all of which are critical for lipid synthesis. Conversely, combined EGF and AngII down-regulated ME1 (malic enzyme 1), which generates NADPH for fatty acid biosynthesis. Although atherosclerosis has been associated with increased lipid accumulation,^45^ the link between altered lipid metabolism in vascular cells and vascular pathophysiology remains unclear. The development and generalization of high-throughput methods such as lipidomics will in future enable characterizing vascular cells lipid signatures associated with pathologies, as recently done for platelets.^46^ Based on these initial results, it is possible that the functional interaction of EGF and AngII regarding lipid metabolism in VSMCs is also primarily protective.

### How do EGF and AngII functionally interact in female VSMCs?

Pharmaceutical inhibition of AT1R by losartan helped to assess if AT1R is involved in the functional interaction between EGF and AngII. Losartan prevented the AngII-dependent ERK phosphorylation in VSMCs, suggesting that their AT1R is functional. However, losartan did not block the effect of AngII on glucose and lipid metabolisms in female VSMCs, implying that these aspects were regulated by another pathway.

**Figure 7:**
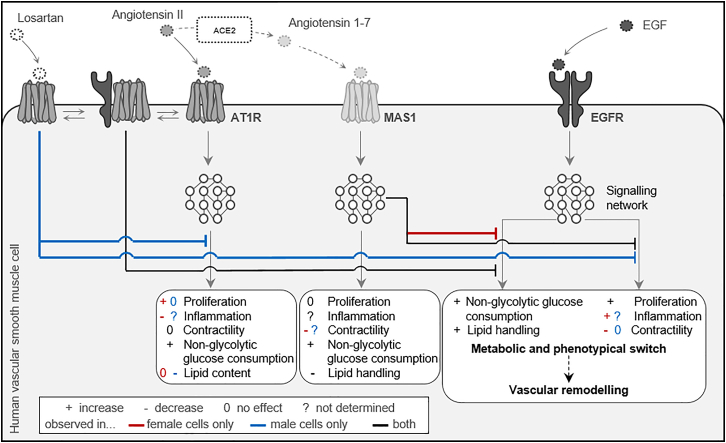
Model of the functional interaction of EGF, AngII, and Ang1-7 in human primary vascular smooth muscle cells

We showed that Ang1-7 triggered effects similar to that of AngII regarding glucose- and lipid-related parameters and also counteracted the effects of EGF. Studies have already reported that AngII and Ang1-7 can have similar effects.^47^^,^^48^ But deciphering the specific events occurring downstream of AT1R or MAS1 remains critical. Indeed, siRNA or gene interference experiments to specifically block one of this pathway (e.g., targeting AT1R or MAS1) have significant disadvantages, especially when subtle effects requiring a well-differentiated phenotype are investigated. In primary cells, viral transfection would be required to achieve the necessary transfection rate. However, this would represent a substantial stress on the cells and probably would substantially affect their differentiated state. Additionally, pharmaceutical intervention is also not straightforward due to the limited availability of MAS1 blockers, which have uncertain inhibitory efficacy.^49^^,^^50^ The selection and testing of a specific inhibitor of MAS1 should be considered in further studies. Nevertheless, in their current state, our results suggest that AngII may partly modulate EGF effects via the ACE2-MAS1 pathway (Figure 7). Contrary to the rather well-documented AT1R-EGFR crosstalk, only little evidence regarding the interaction of EGFR and MAS1 is available yet.^51^^,^^52^ By showing that Ang1-7 inhibits the effect of EGF on both metabolic traits and proliferation, our results highlight the functional interaction of EGFR with an additional GPCR, which could be a new direction for future research.

We also report that losartan surprisingly prevented the effect of EGF on glucose- and lipid-related parameters. We previously showed that the physical interaction of AT1R and EGFR is involved in the membrane-to-nucleus information transfer.^23^ The disruption of this interaction (using AT1R mutants) not only prevents the synergistic response to AngII and EGF in AT1R-transfected HEK293 cells but also partly reduces the effect of EGF itself. This means that a change in AT1R conformation that alters the interaction strength with EGFR may affect associated downstream events. Losartan is considered to have weak inverse agonistic properties compared to other AT1R-blockers^53^ and should induce minimal conformational changes of AT1R. Its binding to AT1R has nonetheless already been shown to result in increasing the interaction strength between AT1R and a dopamine receptor, thereby modulating the downstream effect of the latter.^54^ We propose that the interaction between AT1R and EGFR is also affected by losartan, what ultimately results in preventing the effect of EGF on lipid and glucose handling. This ability to modulate the downstream effects of EGF in VSMCs may constitute a new beneficial aspect of losartan-based therapies. Although it has been described that AT1R conformation is a function of the type of molecule it binds,^55^ we propose that a similar change in interaction strength of the receptors occurs with AngII, which may result in the observed partial negative interaction with EGF (Figure 7).

### The EGF-AngII functional interaction on VSMC metabolism is sex specific

All results discussed hereinabove come from female VSMCs. However, we showed that the negative interaction between EGF and AngII regarding glucose- and lipid-related parameters did not occur in male VSMCs, even if these cells also expressed EGFR and AT1R. These results thus suggest that different interaction mechanisms between EGF- and AngII-associated pathways exist in males and females. Differences between men and women regarding cardiovascular pathophysiology often tend to be attributed to different hormonal status, particularly to estrogen levels.^56^^,^^57^ However, we show here that a “sex memory” is conserved in hormone-free primary culture. These results are in line with previous reports of sex differences in *ex vivo* VSMCs regarding their contractility, proliferative potentials, and resilience to applied stress.^5^^,^^6^^,^^7^ To our knowledge, our study is the first one to report inherent differences regarding intracellular metabolism regulation.

Concerning the mechanisms that drive these sex-related differences, our data show that losartan efficiently prevents the effect of AngII on metabolic traits in male but not in female VSMCs. On the other hand, no functional interaction of EGF and Ang1-7 regarding metabolic traits was observed in male VSMCs, suggesting a limited or even non-existent functional interaction between EGFR and MAS1 in male cells. These results hint that the signal integration at the membrane level already differ between both sex groups. This observation of a more pronounced activity of the Ang1-7/MAS1 pathway in female VSMCs than in male cells is actually consistent with current knowledge. Indeed, reports show that women have higher plasma Ang1-7 concentrations than men,^58^ and female rats have higher intrarenal Ang1-7 concentrations that males,^59^ suggesting that the MAS1 pathway is favored in females compared to males. It has moreover been demonstrated that female rodents express MAS1 receptor more than male ones. These sex-driven differences have not only been associated to sex hormones but also to sex chromosomes,^60^^,^^61^ which is in accordance with the presently used sex hormone-free model. The precise intracellular mechanisms associated with these differences are not elucidated yet and must be considered in the future.

### Clinical implications and perspectives

Our results highlight different aspects that can have clinical implications and support the development of further therapeutic strategies. First, the activation of EGFR induces in a change in the phenotype of VSMCs (more proliferation and inflammation, less contractility) in male and female cells. The effect of EGFR activation is double-edged: it can be beneficial in case of vascular lesions that need repair but can ultimately result in vascular dysfunction if not tightly controlled. Although cancer therapies targeting the EGFR have been associated with cardiotoxicity,^10^ studies in animal models for specific vascular diseases (e.g., congenital vascular anomalies affecting veins^62^ and atherosclerosis^63^) suggest also a beneficial vascular impact of such therapies. Thus, the cardiovascular adverse effects spectrum during cancer therapy with EGFR inhibitors may need a sex-dependent reevaluation. In general, more investigations and clinical consideration regarding the sex-dependent risk-benefits ratio of EGFR targeting therapies in vasculature need to be considered. Our results, which provide more information regarding the role of EGFR in vascular cells, can contribute to this matter.

In addition, our results highlight sex-driven differences in AngII-related pathways, EGFR-GPCR interactions, and responses to losartan. Although losartan has the same antihypertensive effect in men and women,^64^ clinical studies have shown sex-dependent adverse effects^64^ and a milder response to other losartan-based therapies in female patients than in males, especially when it was used for the treatment of heart failure^65^ or left ventricular hypertrophy.^28^ Our study provides here some hints of sex-specific underlying mechanisms (e.g., losartan not preventing the action of AngII and interacting with EGF effect in female cells only) that should be considered for the development of targeted therapies for AngII-related vascular diseases in women. The enhanced activity of MAS1 in female cells shown here must also be considered in this process.

In conclusion, our results show that EGF induces a transdifferentiation of female VSMCs toward a more proliferative and inflammatory phenotype. Although AngII does not appear to modulate this aspect, it counteracts the glucose and lipid metabolic switches triggered by EGF, what can be beneficial for the cells. However, this modulating effect regarding metabolic traits is a sex-specific feature. Further sex-differentiating studies are required to understand better these signaling pathways in vascular cells and to ultimately help define targeted therapies for AngII-related vascular diseases in women.

### Limitations of the study

Our study still presents some limitations. Indeed, the results concerning glucose and lipid handling were generated with cells from various donors and with a high number of independent replicates that guarantee their robustness, but the exact regulated pathways are still to be defined. Furthermore, this study focused on VSMCs. But vessel walls are lined with endothelial cells that constitute an interface between VSMCs and blood vessel content. A similar approach should be applied on endothelial cells or ideally in a more physiological-like bi-layer co-culture system, to grasp fully the effect of EGF and AngII on human vessel walls. Finally, *ex vivo* experiments to assess the vessel contraction potential under the different conditions should be considered, as well as *in situ* studies on human samples, in order to fully translate our results to the clinical situation.

## Resource availability

### Lead contact

Requests for further information should be directed to and will be fulfilled by the lead contact, Virginie Dubourg (virginie.dubourg@medizin.uni-halle.de).

### Materials availability

This study did not generate any new unique reagents.

### Data and code availability

• RNA sequencing data (raw and processed) on which this study is based have been deposited at GEO and are publicly available as of the date of publication (accession number: GSE292207). The whole western blot images are available in the supplementary data.
• This paper does not report original code.
• Any additional information required to reanalyze the data reported in this paper is available from the lead contact upon request.

## Acknowledgments

This project was funded by the 10.13039/501100001659Deutsche Forschungsgemeinschaft (DFG GE 905/24 and BE 3246/6-1).

## Author contributions

V.D., M.G., G.S., B.S., and R.A.B. were involved in the conception and the development of the study design. V.D., N.A., M.K., A.S., and S.M. substantially contributed to the acquisition of the data. R.B. was responsible of the Olink measurement and of the preliminary curation of the data. V.D. and M.G. analyzed and interpreted the data. V.D. drafted the article. M.G., G.S., B.S., R.A.B., and N.A. revised the article. M.G. and R.A.B. acquired the funding. All authors have read and approved the published version of the manuscript.

## Declaration of interests

The authors declare no competing interests.

## STAR★Methods

### Key resources table

**Table:** 

REAGENT or RESOURCE	SOURCE	IDENTIFIER
**Antibodies**		

Rabbit polyclonal phosphor-ERK1/2	Cell Signaling Technology (Germany)	Cat #9101; RRID: AB_331646
Rabbit monoclonal anti-EGR1	Cell Signaling Technology (Germany)	Cat #4154; RRID: AB_2097035
Rabbit polyclonal anti-ACTA2	Cell Signaling Technology (Germany)	Cat #14968; RRID: AB_2798667
Mouse monoclonal anti-BrdU	BD Biosciences (Germany)	Cat #347580; RRID: AB_10015219
Rabbit monoclonal anti-EGFR	Cell Signaling Technology (Germany)	Cat #4267; RRID: AB_2246311
Anti-mouse Alexa Fluor 568	Invitrogen Life Technologies (Germany)	Cat #A10037; RRID: AB_11180865
Anti-rabbit Alexa Fluor 568	Invitrogen Life Technologies (Germany)	Cat #A10042; RRID: AB_2534017
StarBright Blue 520 Anti-Rabbit fluorescent	Bio-Rad (Germany)	Cat #12005869

**Chemicals, peptides, and recombinant proteins**		

Smooth Muscle Cell Growth Medium 2	PromoCell (Germany)	C-22062
Growth Medium 2 Supplement Mix	PromoCell (Germany)	C-39267
EGF (human, recombinant protein)	Merck (Germany)	324831
Angiotensin II (human)	Merck (Germany)	A9525
Angiotensin 1-7	Merck (Germany)	A9202
U46619	Tocris/Bio-Techne (Germany)	1932
Losartan	Merck (Germany)	BP867
BlueZol	Serva (Germany)	39808.01
Turbo DNAse-free kit	Life Technologies (Germany)	AM1907
First strand buffer	Invitrogen (Germany)	Y02321
DTT	Invitrogen (Germany)	Y00147
dNTPs	Thermoscientific (Germany)	R0481
RNAseOut	Invitrogen (Germany)	100000840
RandomPrimer	Invitrogen (Germany)	48190011
SuperScript II Reverse Transcriptase	Invitrogen (Germany)	100004925
QX200 ddPCR EvaGreen Supermix	Bio-Rad (Germany)	1864034
U0126	Merck (Germany)	662005
PMA	Sigma (Germany)	P1585
D-Glucose	Sigma (Germany)	SLBT-4811
4% Formaldehyde	Fischar (Germany)	27279
Triton X-100	Sigma (Germany)	T-9284
BSA	Capricorn (Germany)	BSA-FAF-1U
DAPI	Molecular Probes (Germany)	D-1306
MOPS	Sigma (Germany)	M-3183
Ac-DEVD-AFC	AAT Bioquest, Biomol (Germany)	13401
Trypan Blue	Life Technologies (Germany)	15250061
Pierce BCA Protein Assay	Thermo Scientific (Germany)	23228
Copper(II) sulfate (CuSO_4_)	Roth (Germany)	P024.2
ATP	Sigma (Germany)	A8937
NADP	Sigma (Germany)	N0505
Hexokinase/Glucose-6-phosphate dehydrogenase	Sigma (Germany)	10127825001
Lactate-dehydrogenase	Roche (Germany)	10127230001
NAD	Sigma (Germany)	10127973001
Hydrazine	Sigma (Germany)	225819
Glycine	Sigma (Germany)	*N*-9877
Sodium oleate	Sigma (Germany)	O7501
Sodium palmitate	Sigma (Germany)	P-9767
LipidTOX	Invitrogen (Germany)	H34476
Bromodeoxyuridine (BrdU)	Sigma (Germany)	B5002
Calcein-AM	ATT Bioquest, Biomol (Germany)	ABD-22002
bisBenzimide H 33342 trihydrochloride	Sigma (Germany)	B2261
Ionomycin	Merck (Germany)	I0634
Ponceau S	AppliChem GmbH (Germany)	A2935,0500

**Critical commercial assays**		

RNA 6000 Nano Kit	Agilent Technologies (Germany)	5067–1511
Target 96 Inflammation assay	Olink Proteomics (Sweden)	95302, 95007

**Deposited data**		

Raw and processed data	This paper	GEO database: GSE292207

**Experimental models: cell lines**		

Human aortic smooth muscle cells (female, 63 y.o.)	PromoCell (Germany)	C12533 – Lot n° 416Z048.2
Human aortic smooth muscle cells (female, 51 y.o.)	PromoCell (Germany)	C12533 - Lot n° 437Z016.2
Human coronary smooth muscle cells (female, 32 y.o.)	ATCC (USA)	ATCC-PCS-100-021 - Lot n° 804020
Human aortic smooth muscle cells (female, 12 y.o.)	PromoCell (Germany)	C12533 - Lot n° 454Z019.2
Human aortic smooth muscle cells (female, 38 y.o.)	PromoCell (Germany)	C12533 - Lot n° 431Z013.6
Human coronary smooth muscle cells (female, 43 y.o.)	PromoCell (Germany)	C12511 - Lot n° 495Z001.5
Human aortic smooth muscle cells (male, n.a.)	ProVitro (Germany)	1210614 - Lot n° 296Q130309
Human aortic smooth muscle cells (male, 51 y.o.)	PromoCell (Germany)	C12533 - Lot n° 470Z011.2
Human aortic smooth muscle cells (male, 67 y.o.)	PromoCell (Germany)	C12533 - Lot n° 451Z003.3

**Oligonucleotides**		

Primers for EGFR, ERBB2, AT1R, MAS1 (see Table 3)	This paper	–

**Software and algorithms**		

HISAT	Kim et al.	Version 2.1.0
featureCounts	Liao et al.	Version 2.0.0
R	https://cran-r.project.org/	R 4.0.3
DESeq2 (R package)	https://doi.org/10.18129/B9.bioc.DESeq2	Version 1.28.1
edgeR (R package)	https://doi.org/10.18129/B9.bioc.edgeR	Version 3.30.3

### Experimental model and study participant details

#### Primary cell culture

Commercially available human primary VSMC from multiple donors were used (Table 2). Purity of the isolated cells was assessed by the providers who performed phenotypical characterization. Cells were cultivated at 37°C, 5% CO_2_ in supplemented “Smooth Muscle Cell Basal Medium 2” (5% FCS, 0.5 ng/L EGF, 2 ng/mL FBF, 5 mg/mL Insulin - PromoCell, Germany). Cell synchronization and quiescence were induced by 24 h incubation in media without any supplementation before all experiments. This “minimal” media was also used for further incubation with various substance combinations at the following end concentrations: 10 μg/L EGF (Merck), 10 nM AngII (Merck), 1 μM losartan (Merck), 1 μM Ang1-7 (Merck) or 1 μM U46619 (Bio-Techne). We used cells that underwent no more than 9 cell culture passages for any experiment.

**Table 2:** Information about primary vascular smooth muscle cells used in this study

ID	Sex	Age	Cell type	Provider	(# reference – lot number)	Applications
1	F	51	HAoSMC	PromoCell	(C12533 – 416Z048.2)	RNA sequencing, experimental validations
2	F	63	HAoSMC	PromoCell	(C12533 – 437Z016.2)	RNA sequencing, experimental validations
3	F	32	HCASMC	ATCC	(ATCC-PCS-100-021 - 804020)	RNA sequencing
4	F	12	HAoSMC	PromoCell	(C12533 – 454Z019.2)	experimental validations
5	F	38	HAoSMC	PromoCell	(C12533 – 431Z013.6)	experimental validations
6	F	43	HCASMC	PromoCell	(C12511– 495Z001.5)	experimental validations
7	M	n.a.	HAoSMC	ProVitro	(1210614–296Q130309)	experimental validations
8	M	51	HAoSMC	PromoCell	(C12533 – 470Z011.2)	experimental validations
9	M	67	HAoSMC	PromoCell	(C12533 – 451Z003.3)	experimental validations

Primary human cells isolated from female (F) and male (M) donors were purchased from PromoCell (Germany), ProVitro (Germany), or ATCC (USA). No pathologies were reported for these donors. Two different types of cells were used indifferently; HAoSMC (human aortic [thoracic] smooth muscle cells) and HCASMC (human coronary artery smooth muscle cells).

### Method details

#### RNA sample preparation

Total RNA was isolated after 48 h incubation with EGF and/or AngII with BlueZol reagent as described in the user manual. To remove eventual genomic DNA contaminations, the RNA samples were treated with the “rigorous DNAse treatment” from the “Turbo DNAse-free kit” protocol (Invitrogen, Life Technologies, Germany). All samples were then cleaned by ethanol precipitation (with 3 M sodium acetate, glycogen and 100% ethanol) and the RNA concentration determined by NanoDrop (Biochrom, Germany). RNA 6000 Nano Kit and a 2100 Bioanalyzer System (Agilent Technologies, Germany) was used to assess the quality of the RNA samples (all samples had an RNA Integrity Number (RIN) above 7/10).

#### Bulk RNA sequencing

The whole sequencing procedure was carried out by Novogene Co., Ltd (Cambridge, United-Kingdom), from sequencing libraries preparation with poly(A) enrichment to the paired-end sequencing (2 × 150 bp) runs on an Illumina NovaSeq6000 system (*N* = 11 for each condition, from three different female donors). They also provided the first steps of the analysis, namely adaptor clipping and data quality control. We then took over using HISAT2^66^ (v. 2.1.0) for read mapping to the human genome hg38, and featureCounts^67^ (2.0.0, –M –t exon) to count the mapped reads. The results were annotated using BiomaRt^68^ (v.2.44.4), acessing the Ensembl archive v101. Raw data and processed counts are publicly available on the Gene Expression Omnibus (GEO) database (accession number: GSE292207).

#### Differential expression analysis

Differential expression analysis was performed using DESeq2^69^ (1.28.1) and edgeR^70^ (3.30.3). Based on the results of the principal component analysis that highlighted multiple variables influencing overall gene expression (Figure S8), the study design “∼ replicate + donor + treatment” was applied. The filterByExpr edgeR function and the independent filtering parameter (a = 0.05) of the DESeq2 results function were used to filtered genes with sufficient counts to be included in the statistical analyses without creating biases. The “trimmed mean of M value” (TMM) method was used to calculate normalization factors in the edgeR analysis. Significantly “differentially expressed genes” (DEG) were defined as genes with a false discovery rate (FDR) below 0.05 in both DESeq2 and edgeR outputs (overlap of the respective results), with at least 5 FPM on average in one of the sample groups considered for a given comparison. No amplitude change filtering (log_2_ fold change-based) was applied.

#### Clustering of regulated protein-coding genes

Protein-coding genes identified as differentially expressed were filtered for those regulated either by EGF alone only or by the combination of EGF and AngII only (correspond to the exterior parts of a Venn diagram). The STRING database (https://string-db.org/) and its associated tools were used to identify association networks, as well as functional clusters, among these regulated genes. For each group of genes, a full STRING network was built with a minimum required interaction score set at 0.7 (high confidence). The edge thickness indicates the strength of the data supporting the association between two nodes, and the dotted lines represent edges between clusters. Disconnected nodes are not displayed. “K-means clustering” method served to identify clusters within the network, and enriched Gene Ontology terms and (Reactome-, KEGG-, Wiki-) pathways were automatically returned for each of them.

#### Ingenuity Pathway Analysis

The different lists of regulated genes were uploaded in the Ingenuity Pathway Analysis (IPA)^71^ application (Qiagen, Germany) to perform enrichment analysis. Core analyses were performed in order to predict molecular functions and canonical pathways (filtered to ignore overrepresented cancer-related terms) that may be regulated by these substances, but also to predict which factors (upstream regulators) may be involved in the regulation of these genes. Based on the IPA internal database, predicted activation states were returned for each as Z-scores (positive and negative Z-scores corresponding to putative activation and inhibition, respectively). The outputs of these analyses were aligned with the incorporated “Comparison analysis” tool and the results were extracted before being filtered to identify functions, pathways or upstream regulators that are predicted to be differentially regulated when comparing the effects of EGF alone and when combined with AngII (Figure S9).

#### Reverse transcription and quantification by ddPCR

For cDNA generation by reverse transcription, 1 μg RNA per sample was mixed with the following substances (final concentrations): 1× first strand buffer (Invitrogen), 1.44 mM DTT (Invitrogen), 0.57 mM for each dNTPs (Peqlab), 1.14 U/μL RNAseOut (Invitrogen), 11.5 ng/μL RandomPrimer (Invitrogen) and 2.88 U/μL SuperScript II Reverse Transcriptase (Invitrogen). Samples with similar mix except for the reverse transcriptase were used as negative control during PCR measurements (marked as “(−) RT”). All samples were incubated 5 min at 25 °C, 30 min at 42 °C and 5 min at 95°C in a Biometra T professional thermocycler (Analytik Jena, Germany).

The number of mRNA copies for selected target genes was measured in these samples by digital droplet PCR (ddPCR), using the primers listed in Table 3. All primer pairs had been previously tested by qPCR and the PCR products run on a 2% agarose gel to assure their specificity. The measurement was performed following the instructions provided in the “QX200 ddPCR EvaGreen Supermix” kit (BioRad, Germany). Shortly, oil droplets containing cDNA were generated by a QX200 Droplet Generator (BioRad, Germany), the templates included in these droplets were amplified by PCR (T100 Thermal Cycler, BioRad, Germany) and the droplets containing successfully amplified templates were counted based on their high fluorescence (QX200 Droplet Reader, BioRad, Germany). The number of copies/well of a given template was returned by the software QuantaSoft (BioRad, Germany), and the number of copies/μg RNA was then extrapolated, based on the amount of RNA and the reaction volumes used for cDNA generation.

**Table 3:** Primers used for ddPCR

Target	Intron spanning?	Forward primer (5’ > 3′)	Reverse primer (5’ > 3′)	Annealing temperature
EGFR	yes	GCCAAGGCACGAGTAACAAG	CCACTGTGTTGAGGGCAATG	55
ERBB2	yes	AACCTGGAACTCACCTACCT	AGTTGTCCTCAAAGAGCTGG	60
AT1R	no	TGAACAATAGCCAGGTATCGATCAATG	GGCCAGTGTTTTTCTTTTGAATTT	60
MAS1	no	GGGGTTTGTTGAGAATGGGA	AGGTGGGTGATGTAGACAGT	60

#### Single cell immunofluorescence by in-cell-ELISA

Phosphorylated ERK1/2 was detected at the single cell level by in-cell ELISA in VSMC cultivated in 96-well plates (6 wells per conditions), as previously described.^23^ Shortly, VSMC were incubated for 30 min with different concentrations of AngII (0.1, 1, 10 and 100 nM AngII), 10 μm M U1026 (Merck) or 1 μM PMA (Sigma) in Hepes-Ringer Buffer (122.5 mM NaCl, 5.4 mM KCl, 0.8 mM MgCl_2_∗6 H_2_O, 1.2 mM CaCl_2_∗2 H_2_O, 1 mM NaH_2_PO_4_∗H_2_O, 10 mM HEPES) with 1 mg/mL D-Glucose (Sigma). The incubation solutions were then removed and replaced by 4% Formaldehyde (Fischar) to fix the cells (incubation for 60 min at room temperature). After cell fixation, the cells were permeabilized (for 10 min, with permeabilization buffer: 0.1% Triton X-100 in 1xPBS) and non-specific antibody binding was prevented by incubating them for 1 h at room temperature with a 10% FCS solution (diluted in permeabilization buffer). Anti-phospho-ERK1/2 antibodies (Cell Signaling Technology #9101) were diluted 1:500 in antibody solution dilution (5% BSA (Capricorn) in permeabilization buffer) and added on the cells for overnight incubation at 4°C. Wells incubated with antibody solution but without primary antibody were also included in order to determine the background signal (blank). On the next day, after three 5 min-long washing steps with permeabilization buffer, cells were incubated with anti-rabbit Alexa Fluor 568 secondary antibodies (Invitrogen Life Technologies, #A10042 - diluted 1:500 in antibody dilution solution) in the dark for 1 h and at room temperature. Nuclei were stained by incubation with DAPI (Molecular Probes – final concentration: 2 μg/mL in 1xPBS) for 7 min at room temperature, in the dark as well. Red and blue fluorescence signals (for phospho-ERK1/2 and DNA, respectively) were acquired by digital microscopy (Cytation3 imaging reader, BioTek, Germany). The subsequent analysis was performed with the joined software Gen5 Image Prime 3.08 (BioTek, Germany) to identify the blue objects (single nuclei) and measure the red fluorescence (phospho-ERK) in these objects and in their direct periphery (10 μm wide ring around the blue objects). The amount of phospho-ERK1/2 per well was then defined as (mean of red signal of the blue objects in the considered region – blank) × mean area of the blue objects in the considered region.

#### Caspase-3 activity

Caspase-3 activity was measured after 48 h incubation with EGF and/or AngII in 96-well plates (6 wells per condition), as previously described.^72^ Shortly, the cell culture media was removed and all wells were washed once with 1xPBS before pipetting 30 μL lysis buffer (20 mM MOPS (Merck), 0.1% Triton X-100 (Sigma) in H_2_O, pH 7.4) per well. The plates were incubated 30 min on ice to complete cell lysis. 1.5 μL of caspase substrate Ac-DEVD-AFC (AAT Bioquest) were then pipetted in each well. 30 μL MOPS-Triton and 1.5 μL Ac-DEVD-AFC were also added to wells without cells (blank). The plates were incubated at 37 °C and the fluorescence (excitation/emission wavelengths: 400/505 nm) was measured after 30 and 60 min with a plate reader (Infinite M200, Tecan, Germany). The caspase-3 activity development (slope between 30 and 60 min values corrected for blank) was normalized to the protein content of the concerned well after protein concentration determination as described below.

#### Necrosis measurement by Trypan Blue integration

The necrotic level of the primary cells following incubation with EGF and/or AngII was measured using Trypan Blue integration, as described before.^72^ Shortly, cells were cultivated in 96-well plates and incubated for 48 h with the different substance combinations (6 wells per condition). The cell culture media was removed and all wells were incubated with 50 μL 0.2% Trypan Blue (Life Technologies) for 15 min at 37°C. Wells with no cells but in which Trypan blue was added as well served to determine the background signal (blank). After washing the plates three times with 1xPBS, 100 μL 1% Triton X-100 (Sigma) were added to each well and the plates were incubated for 10 min at room temperature. The absorbance at 560 nm was measured with a plate reader (Infinite M200, Tecan, Germany). Protein concentration was determined for each well as described below so that each Trypan Blue-incorporation value was normalized to the protein content of the well.

#### Protein concentration determination in 96 well-plates

Protein concentration was determined using BCA assay, as previously reported.^72^ After mixing the 50 parts BCA-reagent (Thermo Scientific) with 1 part 4% CuSO_4_, 200 μL were added to each well containing cell lysate (e.g., after caspase or necrosis measurement) or known-protein amounts (standard curve). The plates were incubated for 30 min at 37 °C, and the absorbance measured at 562 nm with a plate reader (Infinite M200, Tecan, Germany).

#### Measurement of inflammation markers

Cell culture media that had been in contact with cells was collected after 48 h incubation under control conditions or with EGF or/and AngII. 92 inflammatory markers were then measured in these samples using the proximity assay-based Target 96 Inflammation assay (Olink Proteomics, Sweden), following manufacturer’s instructions. The measurement was performed at the Institute of Laboratory Medicine, Clinical Chemistry and Molecular Diagnostics, at the University of Leipzig (Germany). After filtering out the markers that were detected in less than 75% of the samples, 36 remained for further analysis. The latter was performed using the PCR C_t_-like “Normalized Protein eXpression” (NPX) unit provided by Olink devices, a relative quantification unit on log_2_ scale that allows the users to identify changes for individual protein levels across the sample sets. If some samples were included in the analysis but still did not reach the detection limit for a given marker, their NPX for this assay was calculated as 0.5× lower detection limit (value provided for each marker individually). The relative amount of each protein *a* was calculated with E_a_ = 2^NPX(a)^, and the relative expression of *a* within each treatment group and for each biological replicate as RE_a_ = E_a,treatment_/E_a,control_. Ratios below 1 were corrected with RE_a_’ = −1/RE_a_ to obtain zero-centred values. Means and 95% confidence intervals were calculated for each marker and each treatment.

#### Glucose consumption and lactate production

We measured the glucose consumption and lactate production by VSMC over their 48 h incubation with EGF and/or AngII. To do so, cell culture media of each well were directly transferred from the 96-well plate used for cell culture to two flat bottom measuring 96-well plates (10 μL in each plate, 6 wells per condition). The two media-containing plates were used to measure glucose and lactate concentrations. For each of them, appropriate standard solutions with known concentrations, H_2_O and medium incubated at 37 °C, 5% CO_2_ for 48 h without cells, were pipetted in remaining empty wells. To the plate including glucose standard solutions, 200 μL of “Glucose-reagent” (110 mM ATP (Sigma), 80 mM NADP (Sigma), 2 U/mL Hexokinase and Glucose-6-phosphate dehydrogenase (Sigma), in TEA Buffer) were added to each well, and the absorbance was measured at 340 nm with a plate reader (Infinite M200, Tecan, Germany) after 15 min incubation at room temperature. For the second plate, containing lactate standard solutions, 200 μL of “Lactate-reagent” (5 mg/mL Lactate-dehydrogenase (Sigma), 40 nM NAD (Sigma), 0.4 M Hydrazine, 0.5 M Glycine) were added to each well, and the absorbance was measured at 340 nm with a plate reader (Infinite M200, Tecan, Germany) after 30 min incubation at 37 °C. The remaining cell culture media was removed from the cell culture plate and, after washing all wells once with 1xPBS, MOPS-Triton was used to lyse the cells. Protein concentration was determined as described hereinabove and was used as cell density estimator to normalize the obtained values. Thereby, glucose consumption and lactate production of the cells were defined as ([Glu]_without cells_ – [Glu]_treatment_)/Protein amount and ([Lactate]_treatment_ – [Lactate]_without cells_)/Protein amount, respectively (with [Glu]_without cells_ ≈ 6 mM and [Lactate]_without cells_ ≈ 0 mM). Their glycolytic index (GI) was defined as GI = produced lactate/2 × consumed glucose, and ΔGI = GI_treatment_ - GI_control_ was calculated for each replicate to evaluate the substance impacts.

#### Neutral lipid content

Cells were split in 96 well plates and incubated with EGF and/or AngII (6 wells per condition). For each condition, half of the wells were supplemented with free fatty acid BSA (16.5 μM) and free fatty acids (FFA) (100 μM Sodium oleate (Sigma) and 100 μM Sodium palmitate (Sigma)). After 48 h incubation, the cells were fixed with 4% formaldehyde (Fischar). After washing the wells twice with 1xPBS, DAPI was added (Molecular Probes – final concentration: 2 μg/mL in 1xPBS) and incubated for 7 min at room temperature. After 3 washing steps with 1xPBS, 50 μL of diluted neutral lipid stain LipidTOX (Invitrogen – 1:200 in 1xPBS) was added to each well and the plates were incubated for 30 min at room temperature. The blue- and red-fluorescence signals (for DNA and lipid, respectively) were acquired by digital microscopy (Cytation3 imaging reader, BioTek, Germany). Using the joined software Gen5 Image Prime 3.08 (BioTek, Germany), blue (nuclei) and red (cells or part of cells) objects were identified. The amount of lipids per well was estimated as follow: (mean red intensity of the red objects × area of the red objects × number of red objects)/number of blue objects. For each condition, the lipid-handling index was defined as the ratio of the amount of lipid in the wells with FFA-supplementation and the amount of lipid in those without FFA.

#### DNA synthesis and cell cycle

Cells were cultivated in 96-well plates and incubated with EGF and/or AngII (6 wells per condition). The evening before reaching 48 h incubation, bromodeoxyuridine (BrdU) was added to each well, at a final concentration of 10 μM (18 h overnight incubation). On the day of the measurement, the cell culture media was removed and cells were fixed with 4% Formaldehyde (Fischar). The plates were washed three times with permeabilization buffer (0.1% Triton X-100 in 1xPBS) and 2 N HCl was added to each well for 30 min at room temperature. After repeating the washing steps with permeabilization buffer, the plates were incubated for 1 h at room temperature with blocking solution (10% FCS in permeabilization buffer). The plates were then incubated overnight with anti-BrdU antibodies (BD Biosciences #347580 – diluted 1:200 in 5% BSA solution). One column of wells was incubated with 5% BSA solution without antibody (blank). On the next day, the plates were washed three times with permeabilization buffer and all wells were then incubated with a red fluorescent secondary antibody (Invitrogen #A10037 - 1:500 in 5% BSA solution) for 1 h at room temperature. After repeating the washing steps, DNA was stained by incubating the plates with DAPI (Molecular Probes – final concentration: 2 μg/mL in permeabilization buffer) for 7 min at room temperature. The plates were washed three times again and then blue- and red-fluorescence signals were acquired by digital microscopy (Cytation3 imaging reader, BioTek, Germany). Images were analyzed with the software Gen5 Image Prime 3.08 (BioTek, Germany). Nuclei were identified (blue objects). Single nuclei DNA content (corresponds to the integral of the blue signal: mean blue fluorescence intensity of the blue objects × area of the blue objects) was used to determine in which cell cycle phase the cells were. The amount of incorporated BrdU (integral of the red signal in the blue objects = (mean red fluorescence intensity of the blue objects - blank) × area of the blue objects) was used as measure of the DNA synthesis. BrdU-positive cells were defined as cells whose nuclei had a mean red fluorescence intensity higher than the red fluorescence background (measured in the “blank” column).

#### Cell proliferation

Images from “DNA synthesis and cell cycle” and “Lipid content” (rows without additional free fatty acids only) were used to estimate the cell proliferation. To do so, cell nuclei (blue objects) were identified and counted in these images with the software Gen5 Image Prime 3.08 (BioTek, Germany).

#### Contractility assay

The cell culture media was removed after 48 h incubation with EGF and/or AngII (in 96-well plate, with 6 wells per condition), and replaced by a staining solution (2 μM Calcein-AM (AAT Bioquest) and 0.5 μg/mL bisBenzimide H 33342 trihydrochloride (Hoechst - Sigma) diluted in 37 °C -warm Hepes-Ringer Buffer (see “in cell ELISA” section) containing 1 mg/mL D-Glucose (Sigma)). The cells were incubated for 30 min at 37 °C. The staining solution was then removed and the plate was washed once with warm HEPES-Ringer buffer with D-Glucose. 50 μL of warm HEPES-Ringer buffer with D-Glucose were then added to each well again and the blue- and green-fluorescent signals (corresponding to DNA and cytoplasm staining, respectively) were acquired with a pre-heated (37 °C) Cytation3 digital microscope (BioTek, Germany). 50 μL of 20 μM ionomycin (Merck – diluted in HEPES-Ringer buffer with D-Glucose) were then added to all wells (final concentration: 10 μM). After 5 min incubation in the pre-heated microscope, the fluorescent signals were acquired a second time. The analysis was performed using the software Gen5 Image Prime 3.08 (BioTek, Germany). Blue and green objects were identified, corresponding to cell nuclei and whole cells, respectively. The latter were filtered for objects containing only one nucleus, an area comprised between 200 μm^2^ and 4000 μm^2^ (to remove identified artifacts and cell clumps) and a length/width ratio above 1.5 (remove additional artifacts and eventual dead and rounding cells). Cell circularity (C) was determined from cell area (A) and perimeter (P) as C = 4 × π × A/P^2^ and was used as a cell shape indicator.^23^ Theoretically, circularity can range from 0 to 1, representing perfectly linear to completely circular morphology, respectively. An acute increase in circularity results from cell contraction. The median circularity of the filtered green objects before the addition of ionomycin was used to determine the initial state of the cells. The ratio of their circularity before and after addition of ionomycin was used to estimate the Ca^2+^-dependent cell contraction.

#### Protein isolation and western blot

Proteins were isolated after 48 h treatment with BlueZol Reagent following the RNA isolation, as described in the user manual. The samples were resuspended in 1% SDS and the concentrations were determined by BCA assay (see “Protein concentration determination” method section). For each sample, 40 μg of proteins were denaturated with 6× Laemmli Buffer for 30 min at 37°C, before being separated by 10% SDS-PAGE. The gel-embedded proteins were transferred onto 45 μm nitrocellulose membranes, which were then stained with Ponceau S solution (AppliChem GmbH, Germany). Pictures of the stained membranes were taken for quantification of the total amount of protein transferred per sample (ChemiDoc MP imaging system from Bio-Rad, Germany). After washing of the membranes with TBS-Tween (17 mM Tris-HCl, 3 mM Tris-base, 140 mM NaCl, pH 7.4 HCl, 0.1% Tween 20), their free binding sites were blocked with a 5% solution of non-fat dry milk in TBS-Tween. The membranes were incubated overnight with primary antibodies against EGFR (Cell Signaling Technology #4267 - 1:500 dilution) diluted in 5% BSA in TBS-Tween. On the next day, after thorough washing with TBS-Tween, StarBright Blue 520 Anti-Rabbit fluorescent secondary antibodies (Bio-Rad #12005869 - diluted 1:4000 in 5% solution of non-fat dry milk in TBS-Tween) and a ChemiDoc MP imaging system (Bio-Rad, Germany) were used for detection of the proteins. ImageLab software (version 6.1, Bio-Rad, Germany) was used for densitometry analysis. Whole protein contents obtained after Ponceau staining were used for normalization before relative quantification of the protein.

#### AT1R blockade

Losartan (Merck) was used when investigating the role of the AngII-receptor AT1R in the observed EGF-AngII crosstalk. Losartan is a clinically used competitive antagonist of AT1R, with an IC_50_ of 20 nM.^73^ In order to ensure a complete AT1-blockade while avoiding nonspecific toxic effects, a final concentration of 1 μM was used for the experiments (50 × IC_50_). Since losartan was solubilized in water, the control condition (cell culture media) served *per se* as negative control.

### Quantification and statistical analysis

#### RNA-sequencing and functional analysis

Differential expression analysis was performed using the gold standard tools DESeq2 and edgeR. The number of biological replicates is indicated in the corresponding method section. For the functional analyses done with GO term enrichment analysis and IPA, the corresponding tests and cut-offs are indicated in the Methods section.

#### Lab experiments

For all experiments performed in 96-well plates, 3 to 6 wells were used by condition (technical replicates) and the mean of the corresponding values were used for further analysis (one plate corresponding to one biological replicate, defined as N in the figure legends). Our experimental design provided strictly connected sets of values for control and treatment conditions, originating from the same donor, at the same cell passage, treated at exactly the same time. This means that each set was biologically independent. This allowed us to calculate the relative effect of each incubation type in a paired manner (relative effect RE_substance,k_ = 100 × value_treatment,k_/value_control,k_, with k ∈ [1-N] and N the total number of biological replicates) (Figure S10). Additionally, the expected additional effect was calculated for the combination of EGF and AngII, with [E + A]_k_ = RE_EGF,k_ + RE_AngII,k_ – 100. Experimental data are presented as mean ±95% confidence intervals. For relative effects, we tested the exclusion of the reference value 100 (corresponding to a difference from controls with α < 0.05). Cells from at least two different donors were used for each experiment depicted here, in order to prevent putative donor-specific effects.

## Supplemental information

Document S1. Figures S1–S10

Table S1. Quantification of inflammation marker secretions by VSMC s Table containing the results of the proximity-based assay for secreted inflammation markers (data used to generate Figure 1E).

Table S2. Results of differential expression and functional analyses The output of the analysis with DESeq2 and edgeR (log_2_ fold change and FDR) are included for each comparison. The table also contains the results of the functional analyses with STRING and IPA.
