# Supplementary material for: Angiotensin II prevents the metabolic but not the transdifferentiating effect of EGF on vascular smooth muscle cells from human female donors
Source: iScience. 2026 Jun 2;29(6):115886. doi: 10.1016/j.isci.2026.115886 (PMC13254892; doi:10.1016/j.isci.2026.115886)
Supplement: Document S1. Figures S1–S10 [file mmc1.pdf]

iScience, Volume *29*

Supplemental information

Angiotensin II prevents the metabolic but not the transdifferentiating effect of EGF on vascular smooth muscle cells from human female donors

Virginie Dubourg, Nasrin Akhtar, Michael Kopf, Amelie Spilker, Sigrid Mildenberger, Barbara Schreier, Gerald Schwerdt, Ronald Biemann, Ralf A. Benndorf, and Michael Gekle

Supplementary Figure S1 - Ex-vivo primary human VSMC express EGF- and AngII-receptors and show responsiveness to AngII. (A) Expression of ERBB family members (including EGFR, also known as ERBB1) and AngII-related receptors in the sequenced control samples (mean FPM ± CI). Expression levels of (B) EGFR, ERBB2, (C) AT1R and MAS1 were measured by ddPCR in a mix of samples already sequenced and prepared independently. Since the primers used for AT1R and MAS1 were not intron-spanning, “cDNA” samples prepared without reverse transcriptase (negative control, noted “(-) RT”) were included as well to exclude the possibility that the number of copies detected were in fact artefacts due to contamination with genomic DNA. (N = 12-15, 3 donors). (D) The data are display here donor-wise (internal donor ID shown at the top e.g. AS1F for first donor, AS2F for 2nd donor...). (E) ERK1/2-phosphorylation level was measured by in cell-ELISA after 30min incubation with increasing concentrations of AngII or EGF(N = 6, 2 donors), and (F) after 30min incubation with 10 µM U1026 (inhibitor of MEK, the mitogen-activated protein kinase kinase, an upstream activator of ERK) or 1 µM PMA (phorbol 12-myristate 13-acetate, served as positive control). Obtained values (normalized to control, here symbolized by a dotted line) define the response range of the primary VSMC regarding ERK-phosphorylation, and indicate that these cells inherently have a relatively high ERK activation level. Incubation with 10 µg/L EGF led to similar results to PMA. (N = 2, n = 5-6) Example pictures are shown in (G) The scale bars correspond to 200 µm. (H) ERK1/2 phosphorylation was also measured after 30min incubation with 1 µM Losartan (specific inhibitor of AT1R) or with 1 µM Losartan and 100 nM AngII. The values obtained with the latter were normalized to those obtained with Losartan alone (reference condition in this case). (N = 6)

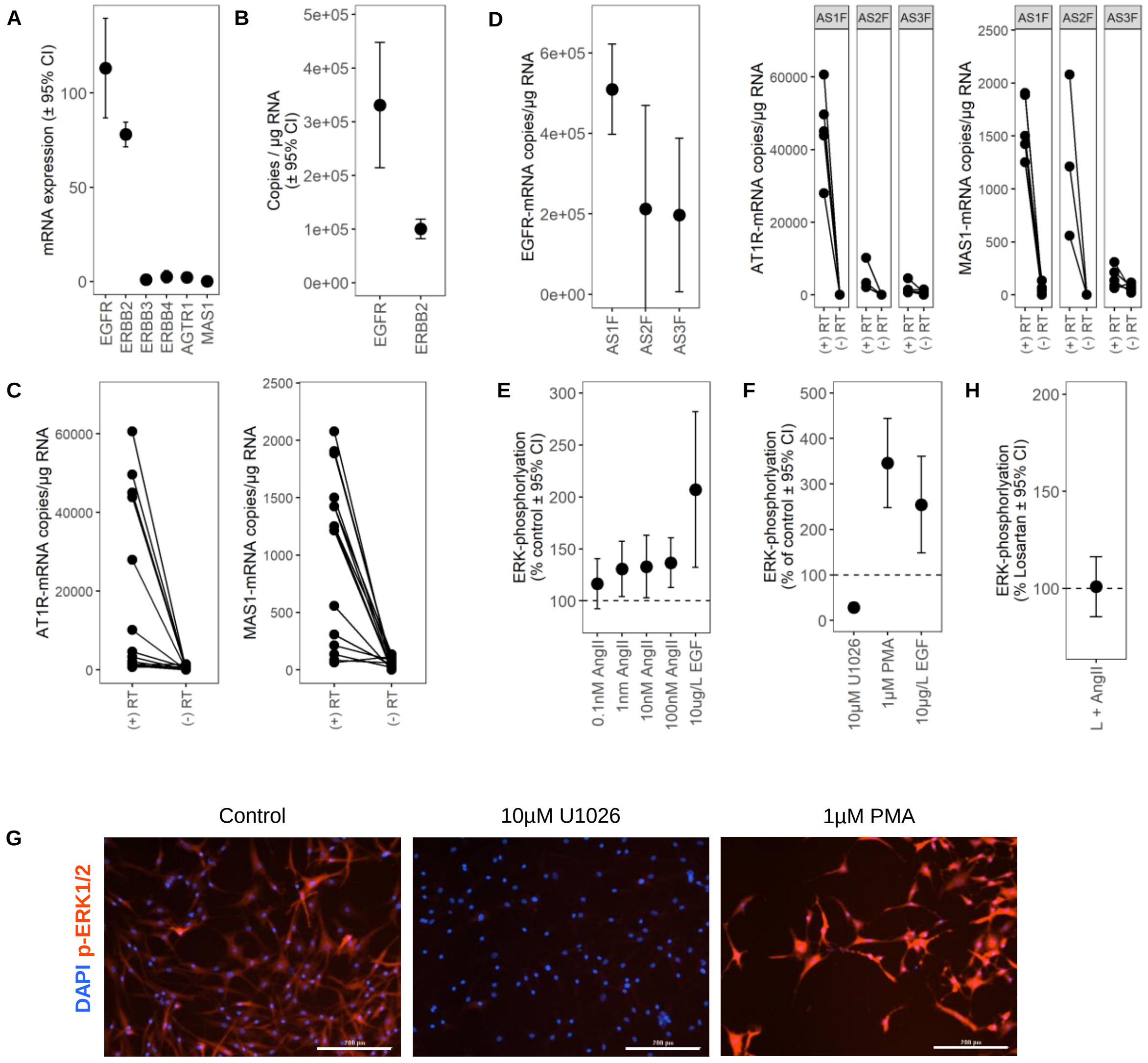

Supplementary Figure S2 – Cardiovascular-related G-protein coupled receptors are lowly expressed.
Li et al (Front. Cardiovasc. Med., 2023) reviewed G-protein coupled receptors (GPCR) involved in cardiovascular diseases, including the angiotensin-related receptors AT1R (or AGTR1) and MAS1. Many of those were not detectable in our RNA-sequencing dataset (grey dots). The expression level of the detectable GPCR (black dots) are displayed (mean FPM in control samples ± 95% CI).

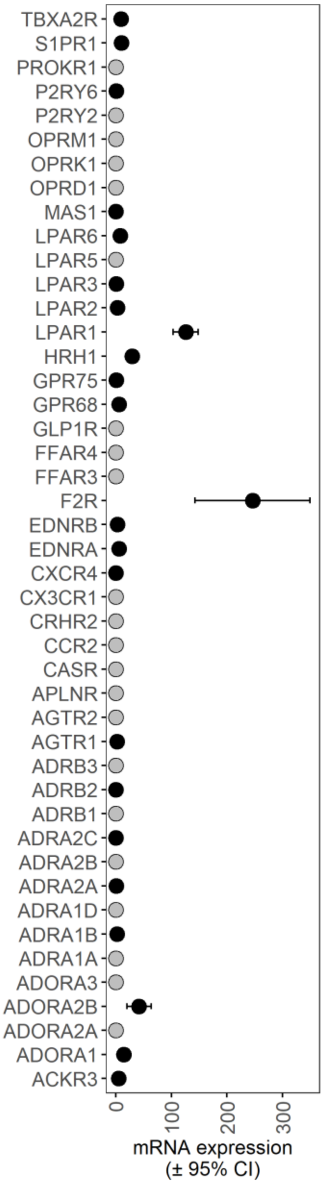

Supplementary Figure S3 – EGF induces a phenotypical switch in female VSMC

In order to verify if eventual changes in cell proliferation were not caused by changes in cell death regulations, we used (A) caspase-3 activity and (B) trypan blue incorporation to estimate the apoptotic and necrotic status of the cells, respectively. Using cells incubated with FCS as a positive control, cell proliferation was then estimated by (C) counting the number of nuclei and (D) measuring the BrdU-incorporation. (E) The proportion of cells contained in the different phases of the cell cycles compared to control conditions and (F) the percentage of BrdU-positive cells in each of these phases following FCS-incubation were also calculated. (G) The cell shape after 48h incubation with the different incubation types was estimated using cell circularity. (H) The ratios of the cell circularity reached after incubation with ionomycin and of the initial cell circularity show that all cells were capable to contract to a certain extend. (I-J) EGF led to the down-regulation of the differentiation and contractile markers ACTA2 and CNN1 (mean FPM in control samples of RNA-sequencing data ± 95% CI). The addition of AngII did not influence these results. (K) On the contrary, EGF led to the up-regulation of the proliferation marker EGR1 (mean FPM in control samples of RNA-sequencing data ± 95% CI). AngII also did not influence these results. – C = control, E = EGF, A = AngII, EA = EGF and AngII, [E+A] = calculated expected additive effect for EGF and AngII, U = U46619. The dotted lines in the figures displaying means ± confidence interval (CI) correspond to the reference level (control values set at 100% for each independent biological replicate).

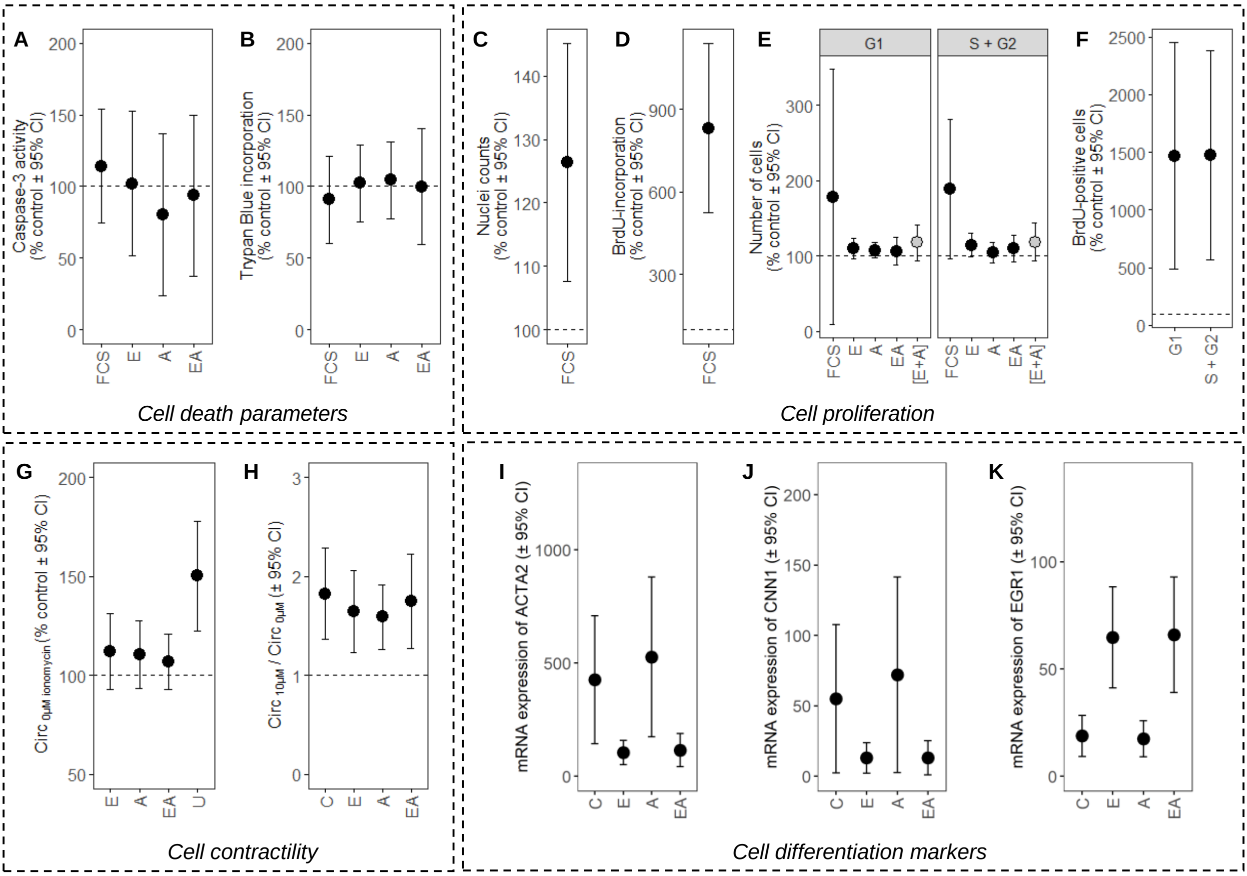

Supplementary Figure S4 – Metabolism regulation

(A) The glycolytic index (GI - ratio of produced lactate / 2 x consumed glucose) was calculated for each condition. A GI of one means that all consumed glucose molecules end up converted into lactate. If GI < 1, it means that some of the glucose products are used to feed other pathways such as the pentose phosphate pathways (PPP) or the TCA cycle within the mitochondria. The same approach was applied (B) after blockade of the AT1R-pathway with Losartan and (C) after the activation of the MAS1 pathway with Ang1-7. (D) The lipid-handling index quantify the overall ability of the cells to use lipids (ratio of the lipid content of cells with and without FFA in the media). A lipid-handling index > 1 means that the lipid content of the cells increases if FFA are present in the media. (E) Incubation with Losartan alone influenced the lipid content of the cells, both in starvation and in FFA-enriched media. The lipid-handling index was nonetheless still increased by EGF. (F) The combination of EGF with Ang1-7 prevented the EGF-induced effect on the cell ability to use lipids. The same experiments were performed on VSMC. (G) and (H) show the GI of the cells under native conditions or with AT1R-blockade, respectively. (I) The experiments were repeated for the combination of EGF and Ang1-7. The lipid handling index was also estimated (J) for the different conditions, (K) after AT1R-blockade and (L) in combination with Ang1-7. – C = control, E = EGF, A = AngII, EA = EGF and AngII, L = Losartan, E+Ang1-7 = EGF+Ang1-7

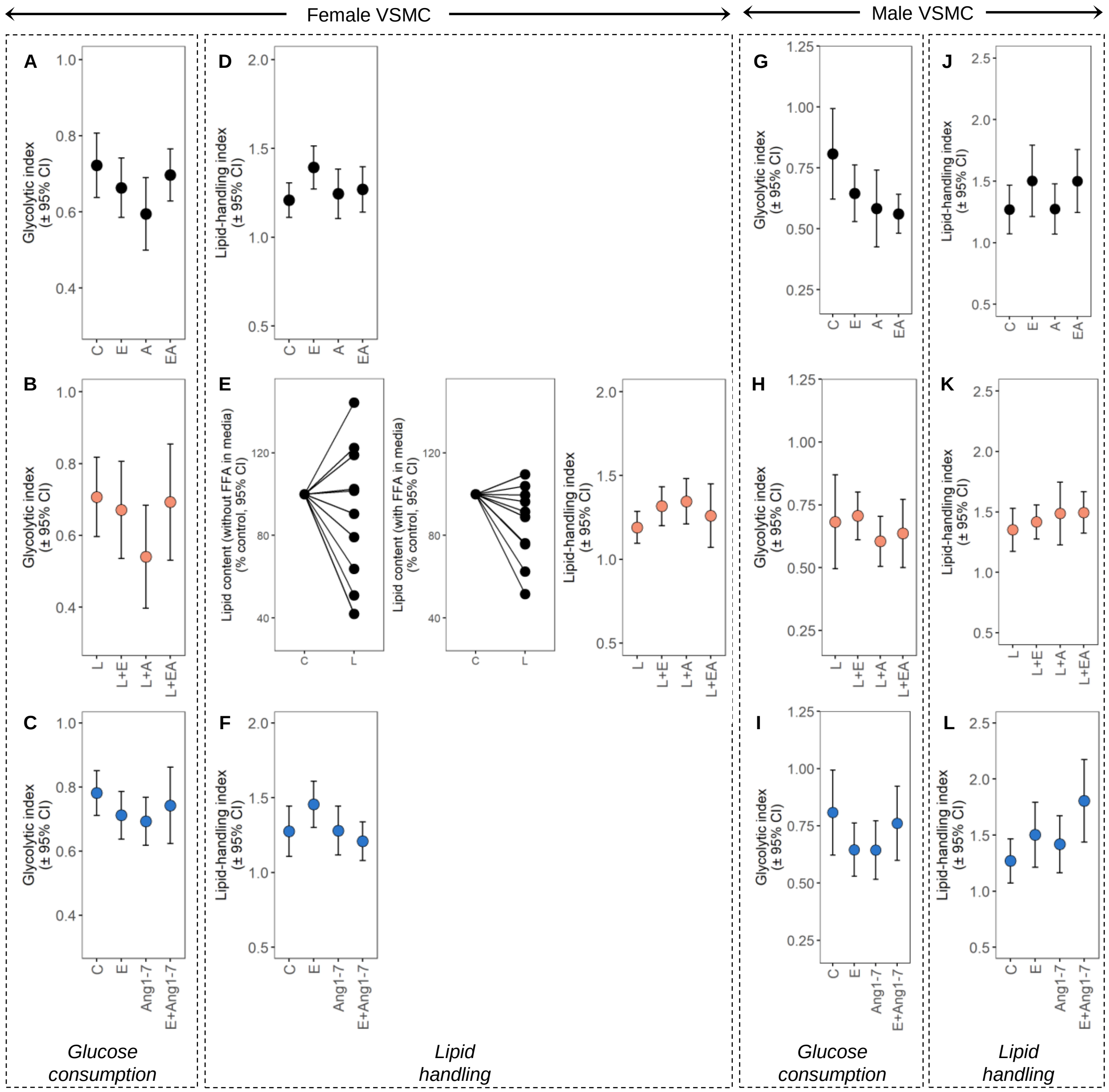

Supplementary Figure S5 – MAS1 activation but not AT1R-blockade influence the effect of EGF on cell proliferation in female VSMC

(A) Expression levels of ACE and ACE2 were measured by ddPCR in order to assess if human primary VSMC had the required enzymes to split AngII into further smaller peptides (N = 6-14, 2-3 donors). (B) Cell proliferation was estimated by counting the number of stained nuclei after 48h incubation. The effects of EGF or AngII were unravel after normalization to the number of nuclei with Losartan alone. (N = 14, 2 donors). (C) Cell proliferation was also estimated after 48h incubation with EGF, Ang1-7 or both. (N = 11, 2 donors). (D) The cell shape after 48h incubation with the different incubation types was estimated using cell circularity. (E) The amplitude of the Ca^{2+}-dependent contraction is returned by the ratio of the cell circularity after adding ionomycin and the initial one. (N = 9, 2 donors). – C = control, L+E = Losartan + EGF, L+A = Losartan + AngII, E = EGF, E+Ang1-7 = EGF and Ang1-7, [E+Ang1-7] = calculated expected additive effect.

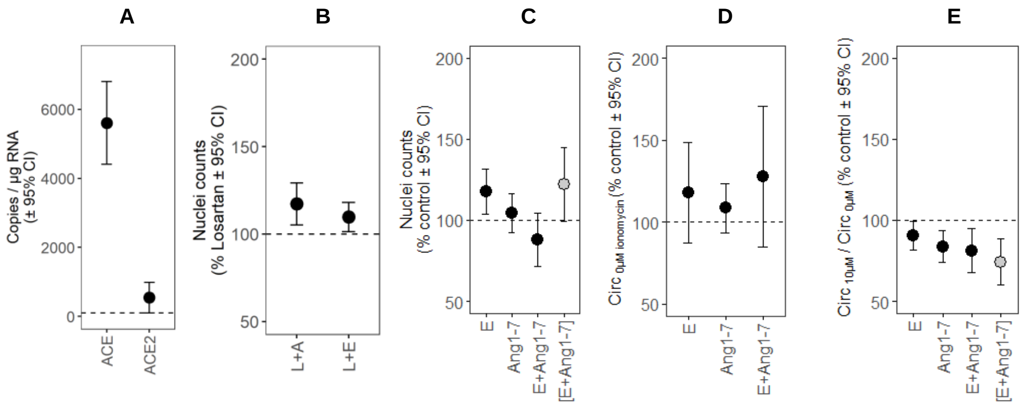

Supplementary Figure S6 – Male VSMC express EGFR and AT1R but at a lower level than female VSMC

Expression levels of (A) EGFR and (B) AT1R were measured by ddPCR in samples from male donors (N = 8, 2 donors). Since the primers used for AT1R were not intron-spanning, "cDNA" samples prepared without reverse transcriptase (negative control, noted "(-) RT") were included as well to exclude the possibility that the number of copies detected were in fact artefacts due to contamination with genomic DNA. The data are display here donor-wise (internal donor ID shown at the top e.g. AS1M for first donor, AS2M for 2nd donor...). (C) The EGFR-expression was also measured at the protein level using Western Blot (N = 4, 2 donors per sex group). Total amounts of transferred proteins (Ponceau) were used for normalization before relative quantification of EGFR (mean expression in male samples used as reference).

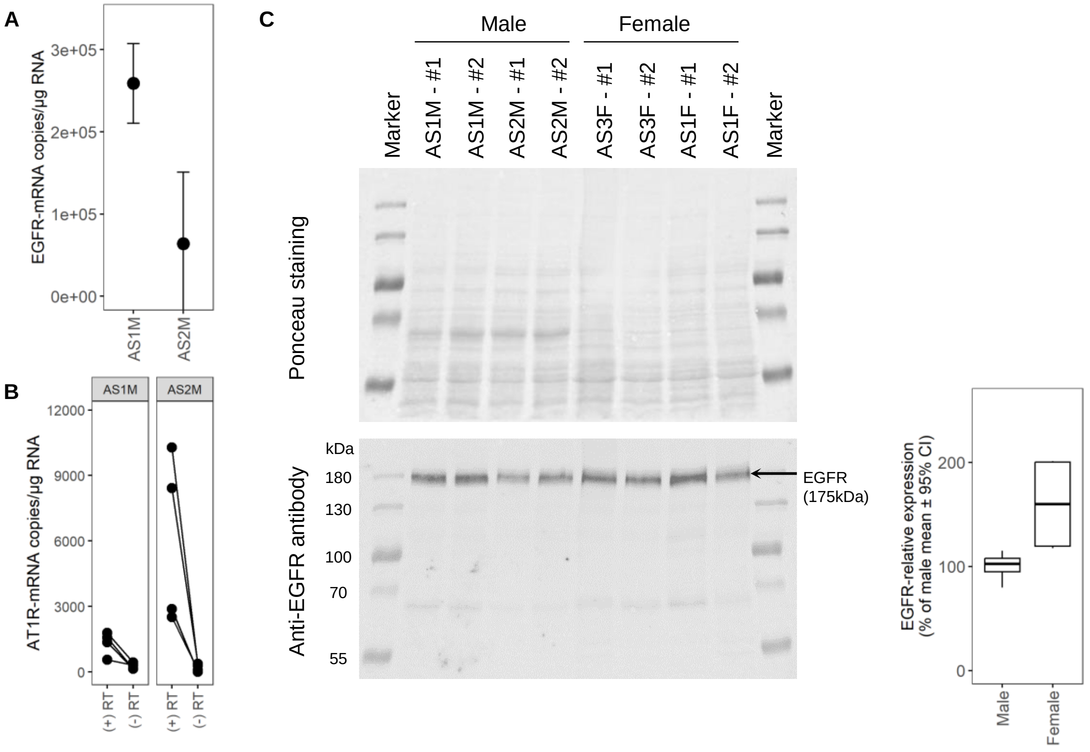

Supplementary Figure S7 – EGF and AngII impact the proliferation but not the Ca^{2+}-dependent contraction of male VSMC

(A) Cell proliferation was estimated by counting the number of stained nuclei after 48h incubation with EGF and/or AngII (N = 19, 2 donors). (B) But these effects were prevented by the addition of Losartan (N = 12, 2 donors). The expression levels of (C) ACTA2, a contractility marker and (D) EGR1, a proliferation marker, were measured by single cell immunofluorescence by in-cell ELISA (N = 12, 1 donor). (E) Cell circularity was used as a proxy for cell shape measurement and the changes of circularity were used as measure of cell contractility. The amplitude of the Ca^{2+}-dependent contraction is returned by the ratio of the cell circularity after adding ionomycin and the initial one. (N = 8, 2 donors). (F) The cell proliferation was also estimated after 48h incubation with EGF and/or Ang1-7 (N = 13, 2 donors). - C = control, E = EGF, A = AngII, EA = EGF and AngII, E+Ang1-7 = EGF and Ang1-7, [E+A] and [E+Ang1-7] = calculated expected additive effects

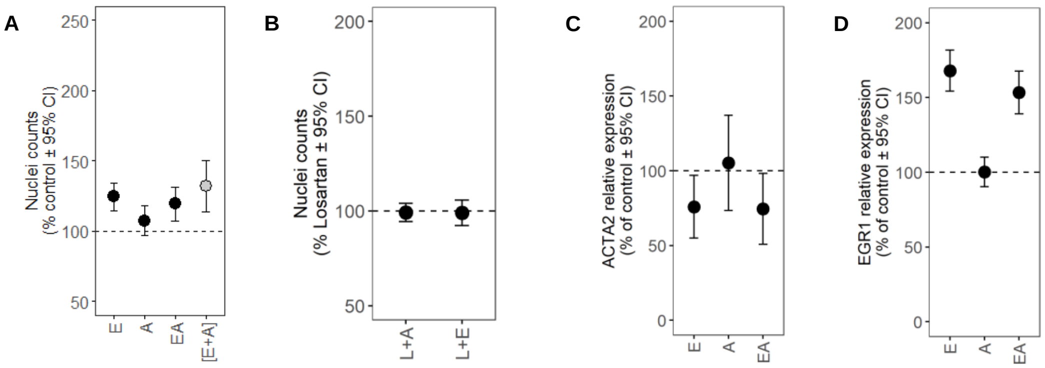

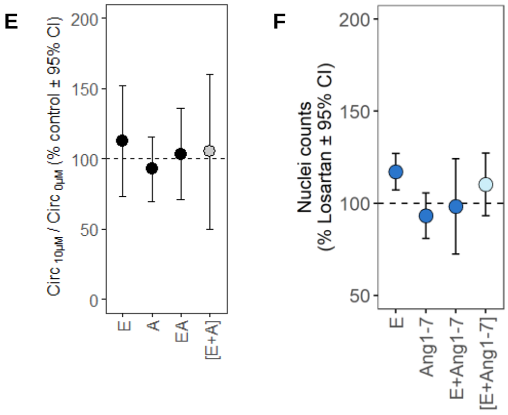

Supplementary Figure S8 – Principal component analysis highlights that the donor origin and the cell passage strongly influence the data

Principal component analysis was performed on RNA-sequencing data using the functions integrated to the DESeq2 R package (see Methods). Each replicate correspond to a different cell passage. – C = control, E = EGF, A = AngII, EA = EGF and AngII

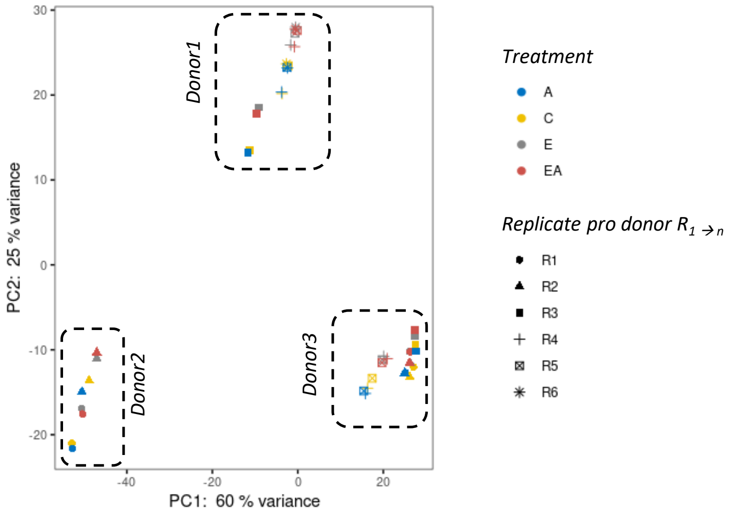

Supplementary Figure S9 - Layering and filtering of the predictions by IPA to identify incubation-specific regulation patterns. (A) The predicted upstream regulators and downstream effects were divided into five upstream- (from 1 to 5) and two downstream-sections (6 and 7), in order to identify different regulation layers. (B) The outputs of the comparisons analyses performed by IPA (alignment of the results obtained for the different lists of regulated genes) were filtered to identify functions and regulators, which are predicted to be differentially regulated when comparing the effect of EGF alone and when combined with AngII. This filtering consisted in going through each of the seven lists (R) of functions, pathways or upstream regulators (see panel A), and for each of these functions or regulators (i) successively comparing the significance (Benjamini Hochberg (BH) p-value) and the predicted activation states (Z = Z scores) for the two incubation conditions (Ri-EGF and Ri-EGF+AngII). For the Z-score comparisons, not only the absolute values are compared but also their signs (sign(Z)) since positive and negative Z-scores correspond to putative activation and inhibition, respectively.

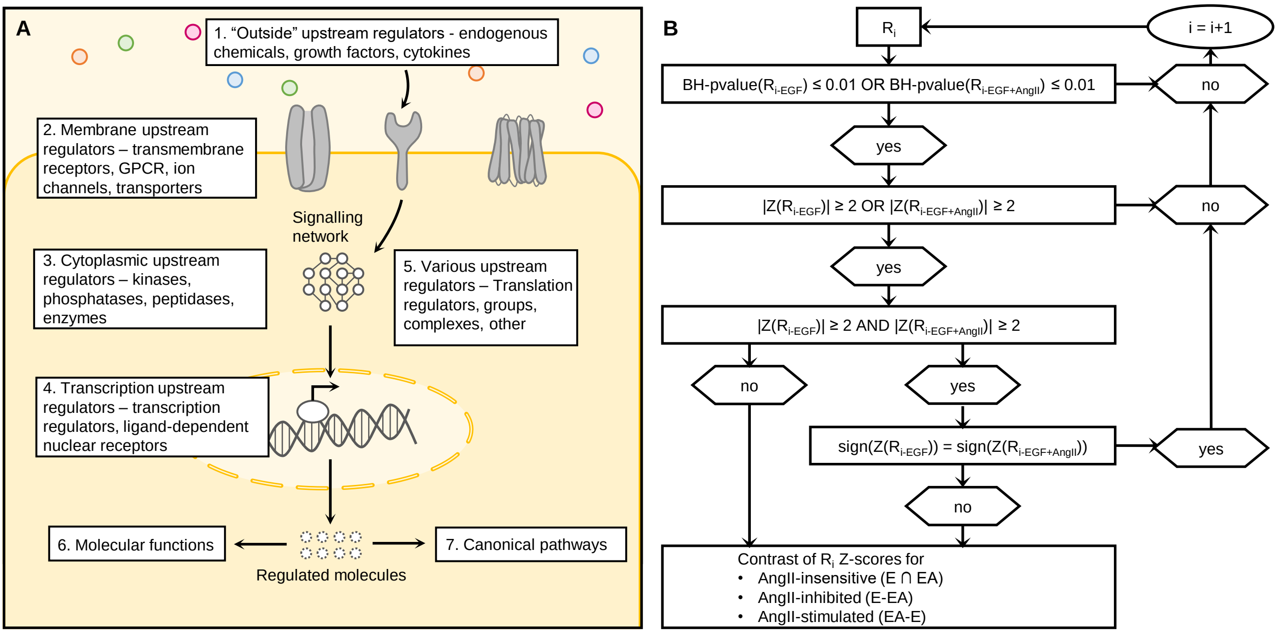

Supplementary Figure S10 – Study design for data generation and analysis. *(next page)*

(A) All our experiments were performed in a strict paired manner, meaning that for each cell culture passage of each donor, we measured all conditions in parallel. This allowed us to calculate the relative effect of each incubation condition.

In case two substances act independently of each other, simultaneous application will lead to an additive effect. In this case, the effect observed by simultaneous application represents the sum of the individual effect ($effect_{substance\ A}$ + $effect_{substance\ B}$ = $effect_{combined\ A\ and\ B}$) and is defined as the "expected additive effect". Here, the expected additive effect of simultaneous application of EGF and AngII (noted [E+A]) was calculated for each replicate, by summing the respective relative effects of EGF and AngII (and subtracting 100 so that the basal level was not considered twice – corresponds to the 100% of the control).

(B) Once all relative (measured or expected) effects were calculated for each replicates, data analysis was performed on all data merged together. The mean for each condition and the corresponding 95% confidence were calculated.

The measured (EA) and expected ([E+A]) effects of simultaneous application of EGF and AngII were compared. If no difference between both was observed, it meant that both substances acted independently, as described above. On the other hand, if the two substances interacted, the measured (EA) and expected ([E+A]) effects differed ($effect_{EGF}$ + $effect_{AngII}$ ≠ $effect_{EGF+AngII}$), with the $effect_{EGF+AngII}$ smaller or higher than the expected additive effect in case of antagonism or a synergism, respectively.

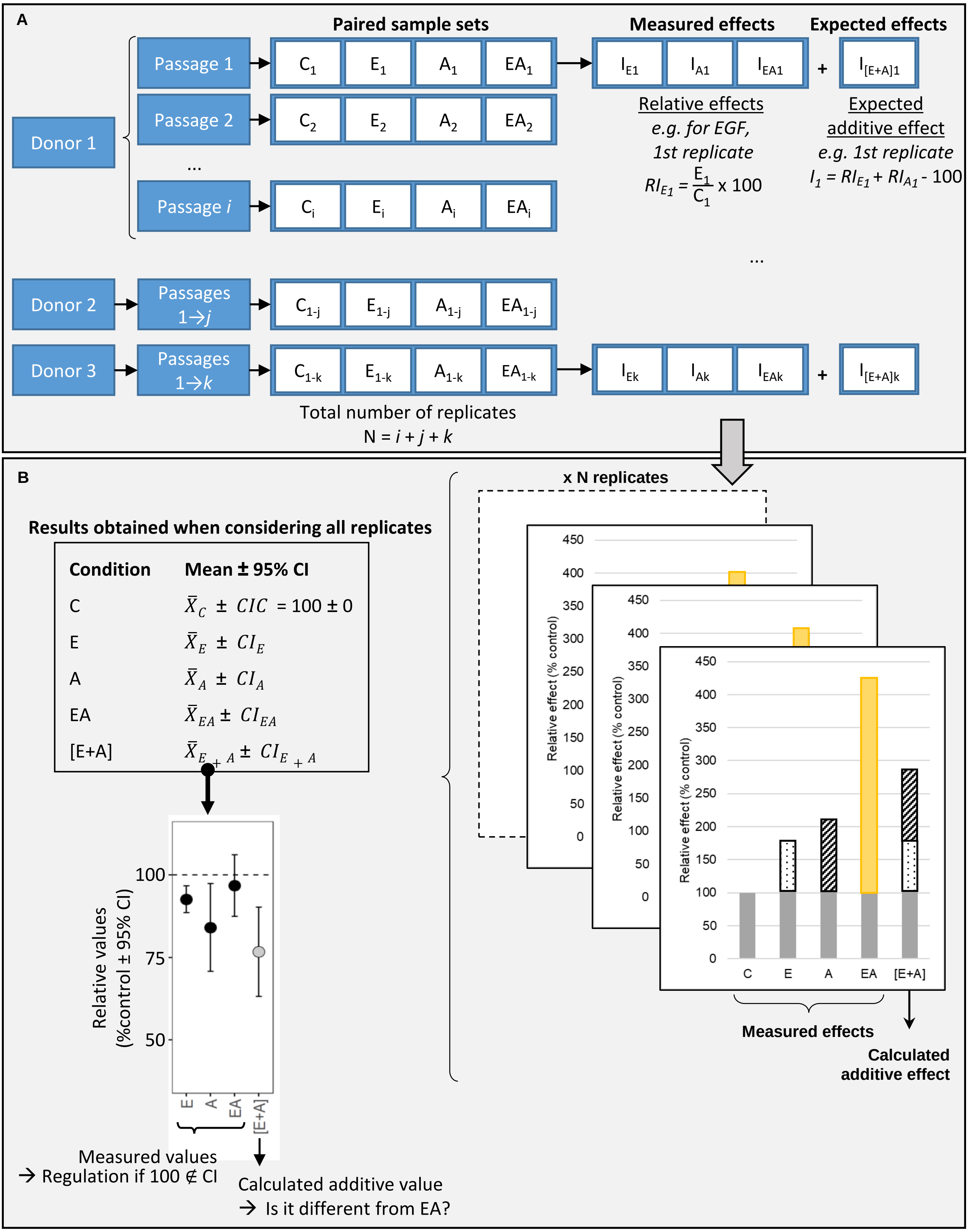

A
Paired sample sets
Measured effects
Expected effects
Donor 1
Passage 1
Passage 2
...
Passage i
C1 E1 A1 EA1
C2 E2 A2 EA2
Ci Ei Ai EAi
IE1 IA1 IEA1
+
I[E+A]1
Relative effects
e.g. for EGF,
1st replicate
RIE1 = E1/C1 x 100
Expected
additive effect
e.g. 1st replicate
I1 = RIE1 + RIA1 - 100
...
Donor 2
Passages 1→j
C1-j E1-j A1-j EA1-j
Donor 3
Passages 1→k
C1-k E1-k A1-k EA1-k
IEk IAk IEAk
I[E+A]k
Total number of replicates
N = i + j + k
B
x N replicates
Results obtained when considering all replicates
Condition
Mean ± 95% CI
C
E
A
EA
[E+A]
= 100 ± 0
Relative effect (% control)
450
400
350
300
250
200
150
100
50
0
C
E
A
EA
[E+A]
Measured effects
Calculated
additive effect
Relative values
(%control ± 95% CI)
100
75
50
Measured values
→ Regulation if 100 ∉ CI
Calculated additive value
→ Is it different from EA?
